# Identification of tryptophan metabolism- and immune-related genes signature and prediction of immune infiltration landscape in bladder urothelial carcinoma

**DOI:** 10.3389/fimmu.2023.1283792

**Published:** 2023-10-26

**Authors:** Guanwen Zhou, Guoliang Qin, Zhaocun Zhang, Haifeng Zhao, Linlin Xue

**Affiliations:** ^1^ Department of Urology, Qilu Hospital of Shandong University, Jinan, China; ^2^ Department of Clinical Laboratory, Shandong Cancer Hospital and Institute, Shandong First Medical University and Shandong Academy of Medical Sciences, Jinan, China

**Keywords:** Bladder urothelial carcinoma, immune, tryptophan metabolism, prognosis, biomarkers

## Abstract

**Introduction:**

Tryptophan metabolism is indirectly involved in immune tolerance and promotes response to anticancer drugs. However, the mechanisms underlying tryptophan metabolism and immune landscape in bladder urothelial carcinoma (BLCA) are not fully understood.

**Methods:**

A BLCA dataset containing 406 tumor samples with clinical survival information and 19 normal samples were obtained from the Cancer Genome Atlas database. The validation set, GSE32894, contained 223 BLCA tumor samples with survival information, and the single-cell dataset, GSE135337, included seven BLCA tumor samples; both were obtained from the gene expression omnibus database. Univariate and multivariate Cox regression analyses were conducted to evaluate clinical parameters and risk scores. Immune infiltration and checkpoint analyses were performed to explore the immune landscape of BLCA. Single-cell analysis was conducted to further identify the roles of model genes in BLCA. Finally, NAMPT expression in BLCA and adjacent tissues was detected using RT-qPCR, CCK-8 and Transwell assays were conducted to determine the role of NAMPT in BLCA cells.

**Results:**

Six crossover genes (TDO2, ACAT1, IDO1, KMO, KYNU, and NAMPT) were identified by overlap analysis of tryptophan metabolism-related genes, immune-related genes, and differentially expressed genes (DEGs). Three biomarkers, NAMPT, IDO1, and ACAT1, were identified using Cox regression analysis. Accordingly, a tryptophan metabolism- and immune-related gene risk model was constructed, and the patients were divided into high- and low-risk groups. There were significant differences in the clinical parameters, prognosis, immune infiltration, and immunotherapy response between the risk groups. RT-qPCR revealed that NAMPT was upregulated in BLCA samples. Knocking down NAMPT significantly inhibited BLCA cell proliferation, migration, and invasion.

**Discussion:**

In our study, we constructed a tryptophan metabolism- and immune-related gene risk model based on three biomarkers, namely NAMPT, IDO1, and ACAT1, that were significantly associated with the progression and immune landscape of BLCA. The risk model could effectively predict patient prognosis and immunotherapy response and can guide individualized immunotherapy.

## 1 Introduction

Bladder urothelial carcinoma (BLCA) is the most prevalent malignancy of the urinary system, with an estimated 573,000 new cases and 213,000 deaths in 2020, making it the tenth most common malignancy worldwide (1). Despite early diagnosis and advanced treatment, BLCA remains the main cause of tumor-related deaths due to its high recurrence and invasiveness (1, 2). Therefore, further research on the complex pathogenesis of BLCA is urgently required.

As an essential amino acid in the human body, tryptophan participates in the regulation of inflammatory responses, oxidative stress, and immune activation through kynurenine metabolism, and plays an important role in the tumor microenvironment and tumor metabolism (3–5). Aberrant regulation of tryptophan metabolism is closely associated with the occurrence and progression of various tumors, including BLCA (6–9). Abnormal regulation of the immune microenvironment also occurs in BLCA (10, 11). An abnormal immune microenvironment can induce the immune escape of BLCA cells by inhibiting the activity of T cells and natural killer cells (10). In recent years, the rapid development of tumor immunotherapy has provided a new method for the treatment of BLCA. Tumor immunotherapy can regulate immune system function and reactivate the ability of the immune system to kill cancer cells, thereby suppressing tumor cell proliferation and invasion. Immune checkpoint inhibitors (ICIs) are new methods for tumor treatment in addition to surgery, chemotherapy, and radiotherapy, and have been approved for the treatment of melanoma, lung cancer, colorectal cancer, and BLCA (12, 13). Abnormal tryptophan metabolism leads to apoptosis and dysfunction of immune cells and induce the formation of an immunosuppressive microenvironment, thereby weakening the therapeutic effect of ICIs (6). Therefore, it is important to elucidate the role of tryptophan metabolism in the progression and the immune landscape of BLCA.

Our study aimed to explore the mechanisms of tryptophan metabolism and immune-related genes in BLCA and construct a risk model. We found that three biomarkers, NAMPT, IDO1, and ACAT1, were significantly associated with the progression and the immune landscape of BLCA. Accordingly, we constructed a tryptophan metabolism- and immune-related gene risk model and divided the patients into high- and low-risk groups. The risk model could effectively predict patient prognosis and immunotherapy response and guide individualized immunotherapy.

## 2 Materials and methods

### 2.1 Data sources

A BLCA dataset was obtained from the Cancer Genome Atlas (TCGA) database, namely the TCGA-BLCA dataset (training set), containing 406 BLCA (tumor) samples with clinical survival information and 19 normal samples. The validation set, GSE32894, containing 223 BLCA samples with survival information, and the single-cell dataset GSE135337, including seven BLCA tumor samples, were obtained from the GEO online database. Furthermore, 61-tryptophan metabolism-related genes were obtained after removing repetitive data using the MsigDB online database (https://www.gsea-msigdb.org/gsea/msigdb/index.jsp). A total of 2991 immune-related genes were retrieved after removing the repetition data based on the ImmPort (http://www.immport.org/), TISIDB (http://cis.hku.hk/TISIDB), and InnateDB (http://www.innatedb.com) databases.

### 2.2 Screening and enrichment analysis of crossover genes

Differentially expressed genes (DEGs) between the BLCA and normal groups in the TCGA-BLCA dataset were acquired using the DESeq2 (v. 1.34.0) (14) package (|Log2FC| > 1 and P. adj< 0.05). Heat and volcano maps of these DEGs were plotted using the pheatmap (v 1.0.12) and ggplot2 (v 3.3.5) (15) packages, respectively. Furthermore, 61 tryptophan metabolism-related genes, 2991 immune-related genes, and DEGs were subject to overlapping analysis to achieve gene crossover. To study the related signaling pathways and biological functions of these crossover genes, KEGG and GO enrichment analyses (P. adj< 0.05) were conducted using the ClusterProfiler (v. 4.6.0) package (16).

### 2.3 The construction, evaluation, and verification of the risk model

Univariate Cox analysis was performed on the above crossover genes to identify the candidate genes related to prognosis (HR ≠ 1, P< 0.2) (17, 18). Subsequently, the LASSO algorithm was implemented for the candidate genes to identify biomarkers (model genes). Based on the expression of the above biomarkers, a risk model was created and the samples in the training set, TCGA-BLCA, and validation set, GSE32894, were classified into high- and low-risk groups, respectively, using the optimum cut-off value of the risk score.


$$Riskscore_{sample}=\sum_{n=1}^{n}(Coef_{i}*x_{i})$$

Kaplan-Meier (K-M) survival curves and receiver operating characteristic (ROC) curves (1-, 3-, and 5-year) were plotted. Differences in risk scores between the different clinical indicator subgroups (invasion, sex, T stage, M stage, N stage, Age, Grade, and Stage) were analyzed using the Wilcoxon test (P< 0.05).

### 2.4 Independent prognostic analysis

The prognostic value of clinical survival prediction was evaluated by combining risk scores with other clinical features. Clinical features (invasion, age, etc.) and risk scores were included in univariate Cox analysis. Multivariate Cox analysis was implemented for clinical features acquired by univariate Cox analysis to determine independent prognostic factors (P< 0.05). Furthermore, a nomogram was created to predict the survival rates of patients with BLCA (1-, 3-, and 5-year survival rates). Calibration curves and decision curve analysis (DCA) were used to verify the nomogram’s validity.

### 2.5 Immune microenvironment analysis

The CIBERSORT algorithm was used to determine the proportions of immune cell infiltrates in each sample. Differential immune cells between the two subgroups were compared (P< 0.05). The relationships between the differentially expressed immune cells were analyzed using Spearman’s method. The relationships between biomarkers and differential immune cells were computed using the Spearman method. Moreover, the expression differences of 48 immune checkpoints (IDO1, CD27, PDCD1, etc.) between the two risk subgroups were compared. Associations between differential immune checkpoints and biomarkers were computed using Spearman’s method. The Tumor Immune Dysfunction and Exclusion (TIDE) algorithm was used from the TIDE online database (http://tide.dfci.harvard.edu/) to detect dysfunction and exclusion scores. Immunophenoscore (IPS) was calculated based on the gene expression of representative cell types using the TCIA database (https://tcia.at/). Moreover, the differences in the TIDE and IPS scores between the two subgroups were compared.

### 2.6 Mutation analysis

In this study, we used the maftools (v 2.10.5) package (19) to analyze the tumor mutation burden (TMB) in the two risk subgroups. In TCGA-BLCA dataset, mutations in IDO1, IDO2, and TDO2 between the two risk subgroups were analyzed. Furthermore, the BLCA samples in TCGA-BLCA dataset were divided into four groups: high TMB-high-risk, low TMB-high-risk, high TMB-low-risk, and low TMB-low-risk. K-M survival curves for the four subgroups were plotted.

### 2.7 Single-cell analysis

In this study, we used the Seurat (v 4.1.0) package for the quality control of the GSE135337 dataset. First, cells with less than 200 genes, genes included in less than three cells, and cells with expressed genes fewer than 100 or more than 5000 were excluded, the proportion of mitochondria genes was limited to less than 5%. The ‘Normalize Data’ and ‘Find Variable Features’ functions were used to standardize the data. Principal components analysis (PCA) was conducted using the ‘JackStrawPlot’ function. The cells were clustered using uniform manifold approximation and projection (UMAP) (resolution = 0.4). The cell groups were annotated using marker genes (PDPN, TAGLN, PECAM1, EPCAM, CD3E, DCN, KRT8, CD2, KRT18, CD14, CSF1R, AIF1, VWF, CD3D, and CLDN5) (20). Subsequently, the expression levels of the three biomarkers in different cell groups were analyzed and visualized.

### 2.8 Patient samples

BLCA and paracancerous tissues were obtained from patients with BLCA at the Qilu Hospital of Shandong University between 2021 and 2022. All participants were informed of the study before surgery and provided consent. This study was approved by the Institutional Review Board of the Qilu Hospital of Shandong University (No.2020046).

### 2.9 Cell culture

BLCA cell lines, T24 and 5637, were purchased from the Type Culture Collection of the Chinese Academy of Sciences (Shanghai, China). All cell lines were tested for mycoplasma and resulted negative. T24 and 5637 were cultured in 1640 medium (Gibco, USA) supplemented with 10% fetal bovine serum (FBS, Gibco, USA). All cell lines were cultured in a 5% CO2 incubator at 37°C.

### 2.10 siRNA transfection

Cells were plated in six-well dishes and transfected with siRNA-NAMPT or negative control using Lipofectamine 3000 (Invitrogen, USA). All siRNA sequences are listed in **Supplementary Table 1**.

### 2.11 RNA isolation and quantitative reverse transcription polymerase chain reaction (RT-qPCR)

Total RNA was extracted from tissues and cell lines using the TRIzol reagent (Invitrogen, USA). cDNA was synthesized from the total RNA using Evo M-MLV RT Premix (Accurate Biology, China). RT-qPCR was performed using a Premix Pro Taq HS qPCR Kit (Accurate Biology, China) on a LightCycler 96 instrument (Roche, Basel, Switzerland). β-actin was used as an internal control. All assays were replicated three times, and the data were analyzed using the 2−ΔΔCT method. All PCR primers were purchased from Accurate Biology (Shanghai, China), and sequences are listed in **Supplementary Table 2**.

### 2.12 Cell Counting kit-8 (CCK-8) and transwell assays

Cells were seeded in 96 well plates at a density of approximately 2000 cells per well. Cell Counting kit-8 (CCK-8) (Bioss, China) was used to detect cell proliferation at 0, 24, 48, 72, and 96 h after culture. Absorbance was measured at 450 nm using a spectrophotometer (Tecan, Switzerland).

For Transwell assay, cells were seeded into an 8.0 Corning™ 24-well Transwell assay plate (Corning, USA) at a density of approximately 20,000 cells per well. After 24 h in an incubator with 5% CO2 at 37°C, the cells below the membrane were fixed with methanol and stained with crystal violet. The cell numbers in three random fields were counted.

## 3 Results

### 3.1 A total of six crossover genes were acquired by crossing tryptophan metabolism-related genes, immune-related genes, and DEGs

**Figure 1:**
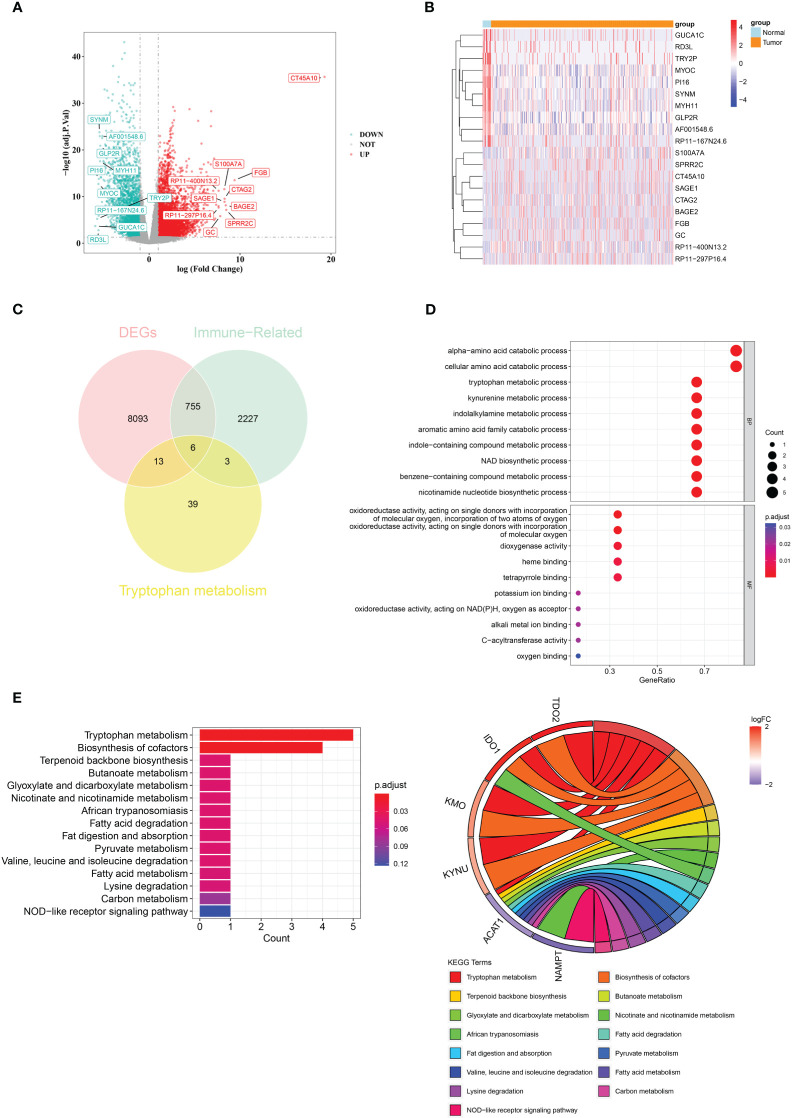
A total of six crossover genes were acquired through the overlap of tryptophan metabolism-related genes, immune-related genes, and DEGs. **(A)** Differentially expressed genes (DEGs) between the BLCA and normal groups. **(B)** The expressional heat map of the top 10 up- and down-regulated DEGs. **(C)** Six crossover genes achieved by intersection analysis. **(D, E)** GO and KEGG analyses of crossover genes.

There were 8867 DEGs between BLCA and normal samples (**Figure 1A**; **Supplementary Table 3**). The expression heatmap of the top 10 upregulated and downregulated DEGs is shown in **Figure 1B**. Through an intersection analysis, using the Venn diagram, six genes (TDO2, ACAT1, IDO1, KMO, KYNU, and NAMPT) were identified (**Figure 1C**). The enrichment analysis results showed that the crossover genes were mainly associated with ‘alpha-amino acid catabolic process’, ‘dioxygenase activity’ GO terms, and ‘Tryptophan metabolism’, ‘Biosynthesis of cofactors’ KEGG pathways (**Figures 1D, E**; **Supplementary Tables 4, 5**).

### 3.2 NAMPT, IDO1, and ACAT1 are tryptophan metabolism- and immune-related biomarkers for BLCA

**Figure 2:**
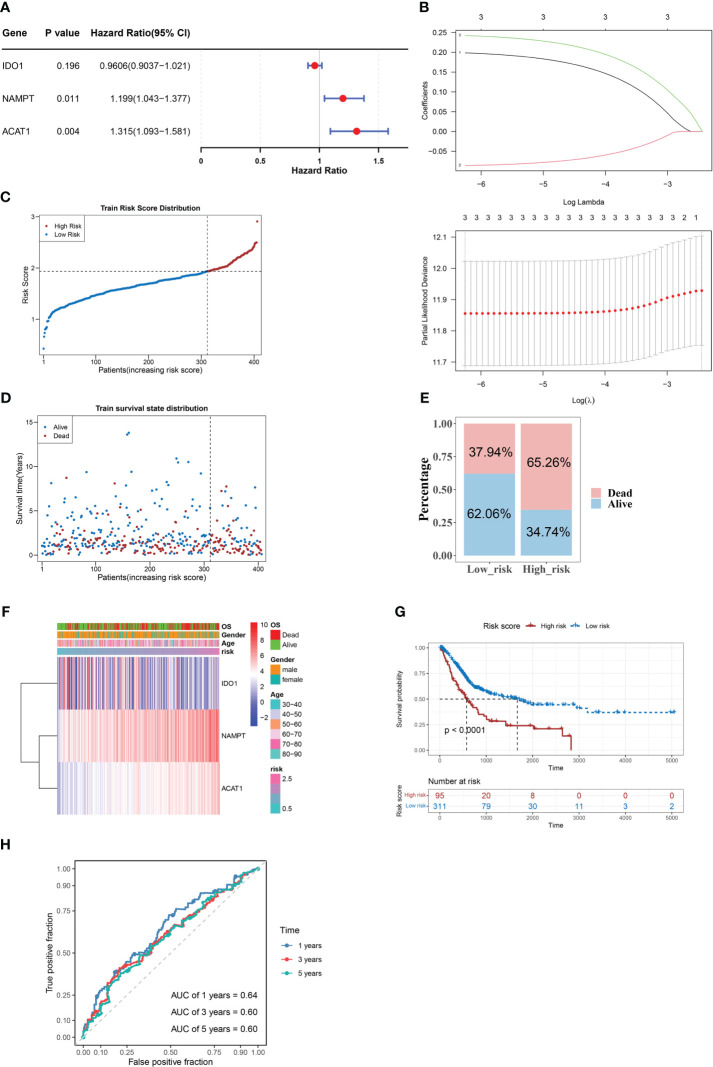
NAMPT, IDO1, and ACAT1 are tryptophan metabolism and immune-related biomarkers for BLCA. **(A)** Univariate Cox analysis of the prognostic candidate genes. **(B)** Average of coefficients of the three biomarkers (NAMPT, IDO1, and ACAT1) in the LASSO Cox regression at each lambda value (above). The partial likelihood deviance varies in accordance with the trend of the log lambda (below). **(C–E)** The distribution of risk scores **(C)**, the distribution of survival status **(D)**, and the proportion of people with different survival statuses **(E)** in the training set. **(F)** Heatmap of the three biomarkers. **(G)** Kaplan Meier curve for the training set. **(H)** 1-, 3-, and 5-year ROC curves for the training set.

Three candidate prognostic genes, NAMPT, IDO1, and ACAT1, were identified by univariate Cox regression analysis (**Figure 2A**). Three biomarkers (NAMPT, IDO1, and ACAT1) were identified using the LASSO algorithm (lambda = 0.001919658) (**Figure 2B**). In the TCGA-BLCA dataset, BLCA samples were classified into two risk subgroups using the best cut-off value of risk score at 1.933147; the proportion of deaths in the high-risk group was significantly higher than that in the low-risk group (**Figures 2C–E**). With an increase in the risk score, we found that the expression of IDO1 was downregulated, while those of NAMPT and ACAT1 were upregulated (**Figure 2F**). We found a distinct survival difference between these two subgroups (P< 0.05), with patients in the high-risk group usually having a poorer prognosis than those in the low-risk group (**Figure 2G**). Moreover, the area under the ROC curve (AUC) values (1-, 3-, and 5-year) were all above or equal to 0.6, suggesting that the risk score could better predict the survival status of BLCA patients (**Figure 2H**).

### 3.3 Accuracy of the predictive model was confirmed using the validation set

**Figure 3:**
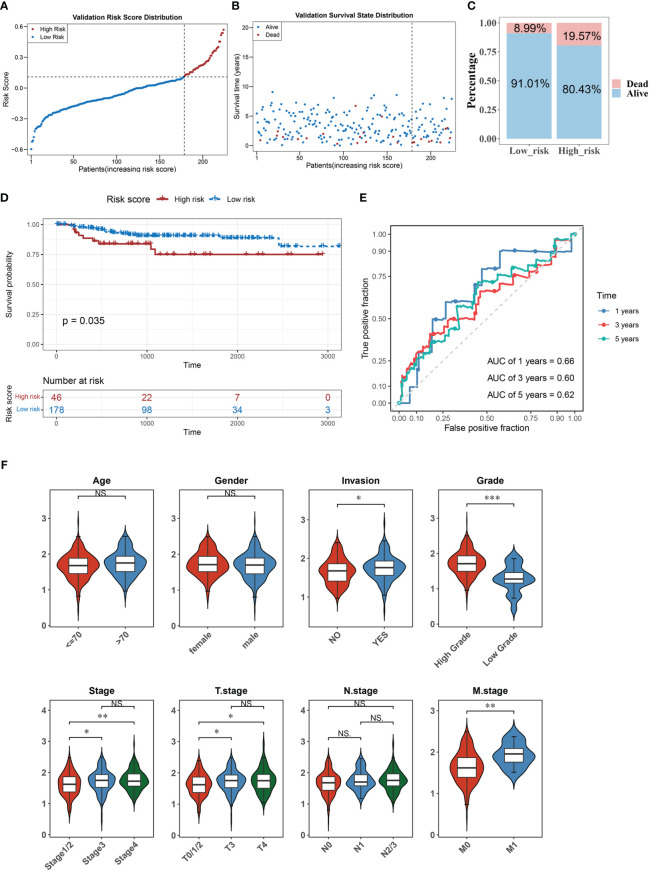
The accuracy of the predictive model was confirmed in the validation set. **(A–C)** The distribution of risk scores **(A)**, the distribution of survival status **(B)**, and the proportion of people with different survival statuses **(C)** in the validation set. **(D)** Kaplan Meier curve for the validation set. **(E)** 1-, 3-, and 5-year ROC curves for the validation set. **(F)** Distribution of risk scores for different clinical features. *P< 0.05, **P< 0.01, ***P< 0.001. . ns, No significance.

We verified the risk model’s utility using the validation set GSE32894 and found that the results were consistent with those of the training set (**Figures 3A–E**). Furthermore, there were significant differences in the corresponding risk scores for invasion (YES and NO), tumor stage (Stage 1/2 and Stage 3, Stage 1/2, and Stage 4), grade (High and Low), T stage (T0/1/2 and T3, T0/1/2, and T4), and M stage (M0 and M1) (**Figure 3F**).

### 3.4 Construction of a nomogram comprising independent prognostic factors (risk score and invasion)

**Figure 4:**
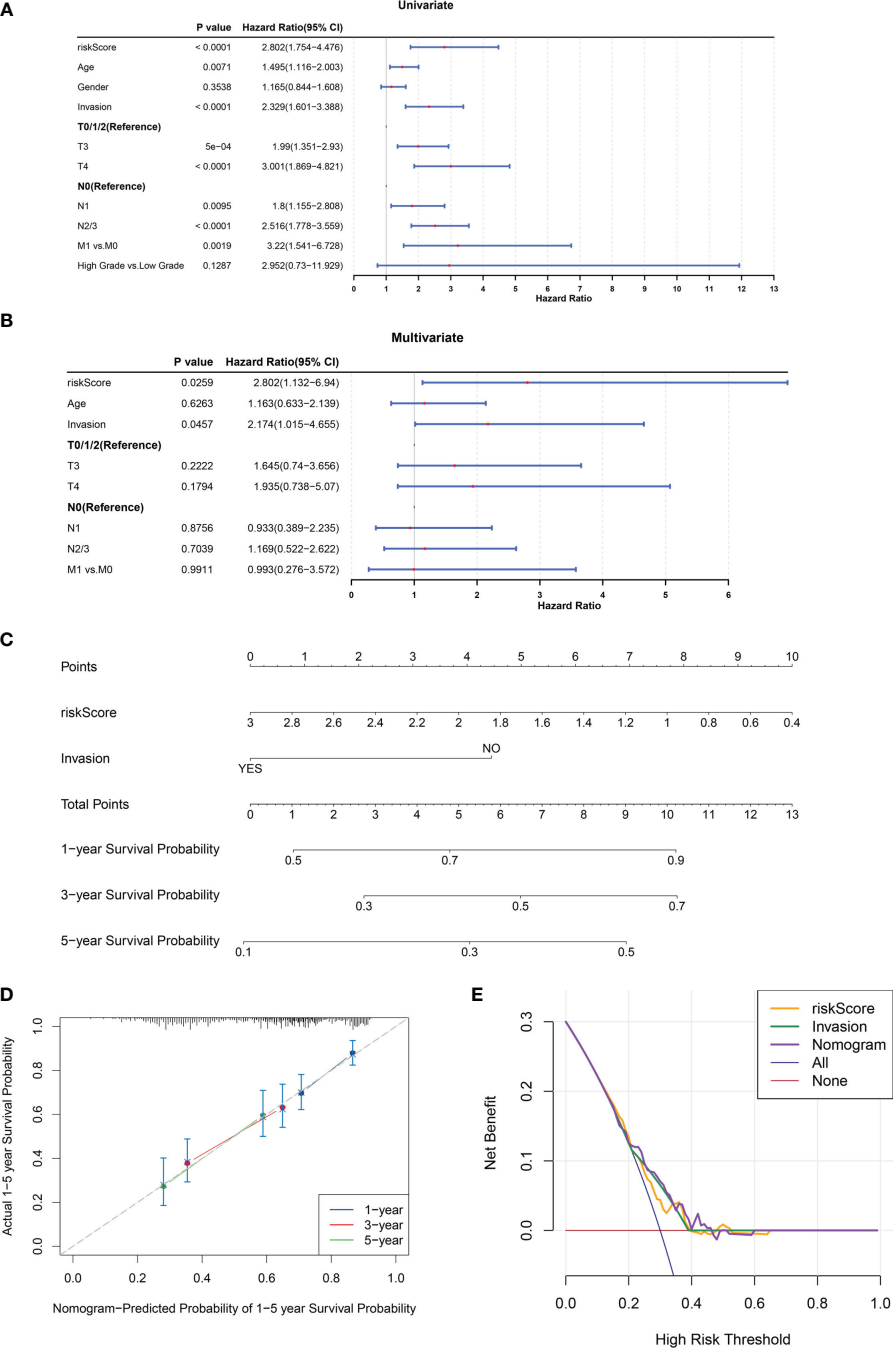
A nomogram comprising independent prognostic factors (risk score and invasion) was created. **(A, B)** Univariate **(A)** and multivariate **(B)** Cox regression analyses of potential prognostic factors for overall survival. **(C)** The nomogram for predicting survival in BLCA patients (1-, 3- and 5-year). **(D, E)** Evaluation of the accuracy of prediction using the calibration curve **(D)** and DCA curve **(E)**.

Univariate and multivariate Cox regression analyses were conducted to evaluate the clinical parameters and risk score to assess their prognostic value. The results demonstrated that risk score, age, invasion, T stage, N stage, and M stage were significant prognostic factors (**Figure 4A**). Multivariate Cox analysis was implemented for clinical features acquired by univariate Cox analysis to determine independent prognostic factors, the results demonstrated that risk score and invasion remained independent prognostic factors for BLCA (**Figure 4B**). Subsequently, a nomogram for predicting survival in patients with BLCA (1, 3- and 5-year) was created based on risk score and invasion (**Figure 4C**). Calibration curves and DCA indicated that the nomogram had a favorable predictive ability for BLCA (**Figures 4D, E**).

### 3.5 Immune infiltration and immune checkpoint analyses for tryptophan metabolism and immune-related biomarkers in BLCA

**Figure 5:**
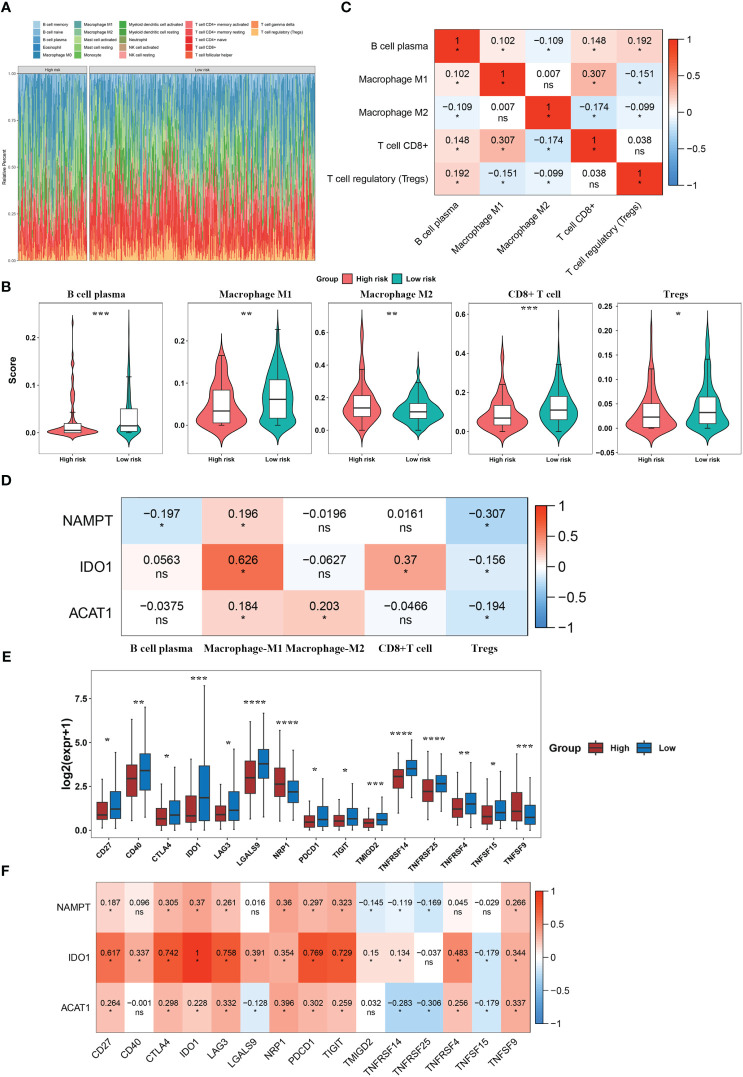
Immune infiltration and immune checkpoint analyses for tryptophan metabolism and immune-related biomarkers for BLCA. **(A)** Immune cell infiltration levels in BLCA patients. **(B)** Differential immune cells between the two risk subgroups. **(C)** Correlation heatmap of differential immune cells. **(D)** Correlation heatmap between model genes and differential immune cells. **(E)** Immune checkpoints with differential expression. **(F)** Correlation heatmap between model genes and differential immune checkpoints. *P< 0.05, **P< 0.01, ***P< 0.001, ****P< 0.0001. ns, No significance.

To clarify the relationship between our prognostic risk model and the tumor immune microenvironment, we investigated the differences in immune cell infiltration between the high- and low-risk groups using the CIBERSORT algorithm (**Figure 5A**). Furthermore, we found five immune cells [CD8+ T cells, macrophage with M1 phenotype, and regulatory T cells (Tregs)] that were differentially expressed between the two risk subgroups (**Figure 5B**). In the BLCA samples, we analyzed the Spearman correlation between differentially infiltrating immune cells and found a significant correlation between CD8 + T cells and macrophage with M1 phenotype (macrophage M1) (|Cor| > 0.3) (**Figure 5C**). Moreover, NAMPT was significantly negatively correlated with regulatory T cells (Tregs) (Cor = -0.307), and there was a significant positive relationship between macrophages M1 and IDO1 (Cor = 0.626) (**Figure 5D**). We found that 15 immune checkpoints (including IDO1, CD27, PDCD1, etc.) were differentially expressed between the two risk subgroups (P< 0.05) (**Figure 5E**). Among these, IDO1 was positively correlated with most differential immune checkpoints and highly positively associated with PDCD1, CTLA4, CD27, LAG3, and TIGIT (Cor > 0.6) (**Figure 5F**).

### 3.6 Analysis of TIDE, IPS, and mutations between the two risk score groups

**Figure 6:**
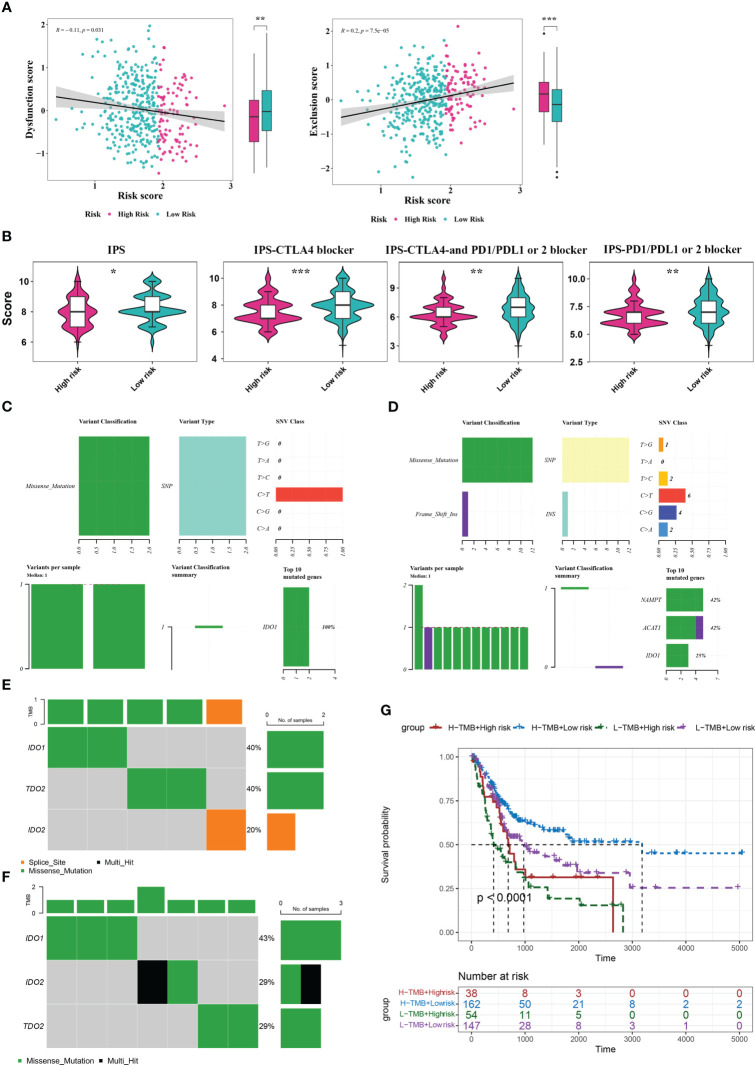
Analysis of TIDE, IPS, and mutations between the two risk score groups. **(A)** TIDE analysis between high- and low-risk groups. **(B)** IPS scores between high- and low-risk groups. **(C, D)** Mutation analysis for genes in the model in the high- **(C)** and low-risk **(D)** groups. **(E, F)** Mutation analysis for IDO1, IDO2, and TDO2 in the high- **(E)** and low-risk **(F)** groups. **(G)** Kaplan Meier curve analysis for the four different groups. *P< 0.05, **P< 0.01, ***P< 0.001.

To explore the guiding value of the risk model for tumor immune exclusion and dysfunction, we used the TIDE algorithm to predict the response to ICIs. The results revealed that the high-risk group had higher exclusion scores but significantly lower dysfunction scores than the low-risk group, suggesting that the high-risk group was more likely to experience T cell exhaustion than the low-risk group, while the low-risk group was more likely to experience immune cell dysfunction (**Figure 6A**). Subsequently, we used IPS to predict the immunotherapeutic response of BLCA patients who received different treatment modalities (such as no treatment, anti-CTLA4, anti-PD1/PD-L1/PD-L2, or combination therapy). We found that patients in the low-risk group had a higher IPS, indicating better immunotherapy efficacy. Thus, these patients were more likely to benefit from immunotherapy (**Figure 6B**).

By analyzing the tumor mutation burden (TMB), we found that only the IDO1 gene was mutated (missense mutation) in the high-risk group, whereas in the low-risk group, NAMTP, ACAT1, and IDO1 were all mutated, most of which were missense mutations and a small fraction were insertion frameshift mutations (**Figures 6C, D**). Three genes (IDO2, IDO1, and TDO2) in the high-risk group were mutated in five samples, whereas these genes in the low-risk group were mutated in seven samples (**Figures 6E, F**). Based on the mutation data from TCGA-BLCA dataset, the BLCA samples in TCGA-BLCA were divided into high TMB-high-risk, high TMB-low-risk, low TMB-high-risk, and low TMB-low-risk groups. K-M curves of the four groups were then analyzed. We found a significant difference in survival among the four groups (P< 0.05), and the survival status was the worst in the low-TMB-high-risk group (**Figure 6G**).

### 3.7 Single-cell analysis for tryptophan metabolism- and immune-related biomarkers in BLCA

**Figure 7:**
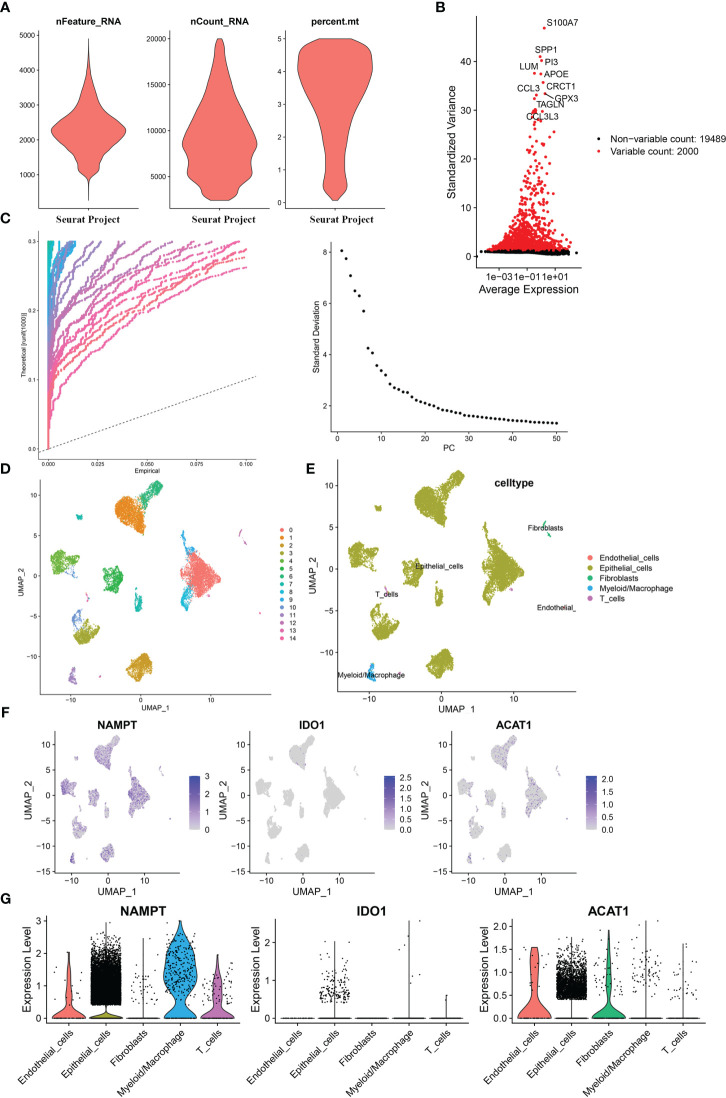
Single-cell analysis for the model genes. **(A)** A total of 18,718 core cells were analyzed after quality control. **(B)** Screening for highly variable gene expression. **(C)** Scatter plots (left) and gravel plots (right) of principal components according to PCA results. **(D)** After UMAP dimensionality reduction, cells were divided into 15 groups. **(E)** Annotation of cell populations using marker genes. **(F)** Expression of model genes in different cell populations. **(G)** Violin plot for expression of model genes in different cell populations.

Based on the single-cell sequencing dataset (GSE135337), 18,718 core cells were acquired after quality control (**Figure 7A**). After normalizing the data, the top 2000 highly variable genes were screened for downstream analysis (**Figure 7B**). According to PCA results, 20 principal components (P< 0.05) were selected for subsequent analyses (**Figure 7C**). Using UMAP, the core cells were clustered into 15 classes (**Figure 7D**). The main annotations included five cell types: endothelial cells, epithelial cells, myeloid/macrophages, fibroblasts, and T cells. We found that NAMPT was expressed in all five cell groups; IDO1 was partially expressed in epithelial cells, myeloid/macrophages, and T cells, and ACAT1 was highly expressed in endothelial cells, epithelial cells, and fibroblasts and partially expressed in myeloid/macrophages and T cells (**Figures 7E–G**).

### 3.8 NAMPT was upregulated in BLCA tissues and could regulate BLCA cell proliferation and invasion *in vitro*

**Figure 8:**
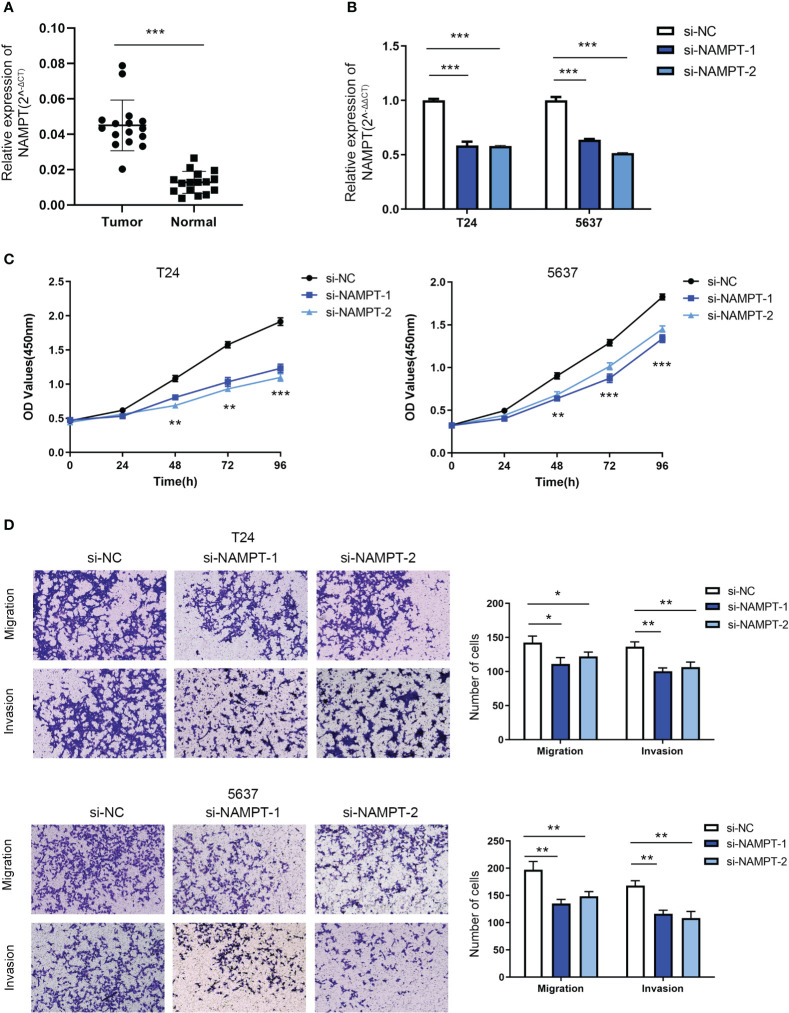
NAMPT was upregulated in BLCA tissues and could regulate BLCA cell proliferation and invasion *in vitro*. **(A)** NAMPT expression in BLCA and matched adjacent normal tissues detected by RT-qPCR. **(B)** Efficiency of NAMPT knockdown was verified by RT-qPCR. **(C)** The proliferative ability of NAMPT knockdown cells was determined using CCK-8 assay. **(D)** Transwell assay was used to detect the migration and invasion abilities of cells with NAMPT knockdown. *P< 0.05, **P< 0.01, ***P< 0.001.

NAMPT expression was significantly higher in cancer tissues than in adjacent tissues (**Figure 8A**). The effects of NAMPT on cell proliferation and invasion were explored to determine its role in BLCA cells. The efficiency of NAMPT knockdown was verified using RT-qPCR (**Figure 8B**). CCK-8 assay demonstrated that NAMPT knockdown significantly inhibited the proliferation of T24 and 5637 cells (**Figure 8C**). Moreover, the migration and invasion abilities of T24 and 5637 cells were significantly decreased after NAMPT knockdown (**Figure 8D**).

## 4 Discussion

BLCA is a common malignancy of the urine system. The incidence and mortality rates of BLCA have increased recently. The 5-year survival rate of muscle-invasive BLCA (MIBC) is less than 50%, and the prognosis of metastatic BLCA is even worse, with a 5-year survival rate of less than 15% (2). Ultrasonography and cystoscopy are traditional methods for diagnosing bladder cancer. In recent years, the researches on bladder cancer biomarkers have received significant attention in order to improve the accuracy of non-invasive detection of bladder cancer. Many cancer-associated molecules have been identified over the recent years which include EGFR, NMP22, FGFR3, p53 etc. (21–23). However, due to the lack of sufficient sensitivity and specificity in most biomarkers, there is still no ideal biomarker in clinical practice that can replace cystoscopy for the diagnosis, treatment, and prognostic evaluation of bladder cancer.

Due to the heterogeneity of BLCA, the response to different molecular subtypes of bladder cancer varies greatly with chemotherapy and targeted therapy. Therefore, there is an urgent need to develop new therapeutic drugs that can significantly inhibit tumor proliferation and effectively improve the prognosis of patients with locally advanced and metastatic BLCA. In recent years, immunotherapy has led to significant breakthroughs in BLCA treatment. Immunotherapeutic drugs such as ICIs have been widely used in BLCA (24). Immune checkpoints are a class of immune regulatory molecules comprising receptors expressed on the surface of immune cells and ligands expressed on the surface of tumor cells. The interaction between these two components can regulate the immune system activity and affect tumor immunity. However, owing to the complexity of the tumor immune microenvironment, the overall effective rate of immunotherapy is only 10– 30%, and most patients cannot benefit from immunotherapy (25). Therefore, improving the responsiveness of patients to immunotherapy and restoring the body’s antitumor immune response are important problems that urgently need to be solved.

In order to adapt to the hypoxic and nutrient-poor microenvironment to achieve rapid growth, tumor cells change their energy metabolism behavior, referred to as “metabolic reprogramming,” basic characteristics of tumors (26). In addition to participating in protein synthesis, tryptophan is an important energy source for the immune system (27). Disorders in tryptophan metabolism can lead to apoptosis and dysfunction of immune cells, induce the formation of an immunosuppressive microenvironment, and affect the efficacy of ICIs. Specifically, abnormal tryptophan metabolism leads to tryptophan depletion, which leads to insufficient energy in immune cells and affects the activity of the immune system (5, 6, 28). Tryptophan depletion leads to increased levels of free tRNAs, which directly activate regulatory T cells (Tregs) through the General Control Non-derepressible-2 (GCN2) pathway, thus inhibiting the activity of antigen-presenting cells and the proliferation of CD8+ T cells (29, 30). Furthermore, the accumulation of tryptophan metabolites enhances IDO1 activity and creates an inhibitory immune microenvironment (6, 31). Therefore, inhibiting the activity of key enzymes (IDO1, IDO2, and TDO) in the tryptophan metabolism pathway can restore the activity of immune cells, which has been investigated as a potential strategy to restore immune function and improve the response to immunotherapy.

Unlike single-gene biomarkers, risk scoring models analyze different model genes together, resulting in higher sensitivity and specificity. Therefore, risk scoring models can be used for early diagnosis, treatment decisions, individual monitoring and follow-up of bladder cancer. Using univariate and multivariate Cox regression analyses, we constructed a risk model that included the NAMPT, ACAT1, and IDO1 genes. The K-M survival and ROC curves were used to verify the validity of the risk model, and the results showed that there was a significant survival difference between the high- and low-risk groups, indicating that the risk model had good predictive ability. NAMPT plays a significant role in various cellular processes, including energy metabolism, nicotinamide adenine dinucleotide (NAD+) biosynthesis, and cell survival (32). High NAMPT expression is associated with enhanced NAD+ biosynthesis, which may lead to advantages in tumor cell proliferation and survival (32, 33). Moreover, an upregulated expression of NAMPT has been associated with chemoresistance and reduced chemotherapy efficacy (34). In this study, we verified that NAMPT knockdown significantly inhibited BLCA cell proliferation, migration and invasion. ACAT1 plays a crucial role in cellular lipid metabolism (35), and several studies have investigated the roles of lipid metabolism and related enzymes in BLCA (36, 37). As a result, there may have potential implications for ACAT1 action in BLCA, although more research is needed to understand its precise role in this context.

Single-cell sequencing allows researchers to study the genetic and genomic characteristics of individual cells, revealing the cellular heterogeneity in tissues and organs. Using a single-cell sequencing dataset, we found that NAMPT and ACAT1 were expressed in various cell types, whereas IDO1 was mainly expressed in immune cells. This further verified that NAMPT and ACAT1 play biological roles mainly by regulating the energy metabolism of various cell types, whereas IDO1 affects tumor progression by regulating the activity of immune cells.

In addition to the tryptophan metabolism pathway, KEGG pathway analysis suggested the enrichment of the NOD-like receptor signaling pathway. NOD-like receptors (NLRs) are a class of signaling receptors found in immune cells that are involved in biological processes such as apoptosis, inflammation, and immune responses (38, 39). Moreover, the NLR signaling pathway can regulate tryptophan metabolism (40), which is involved in the immune response and plays a critical role in tumor immune evasion.

Abnormalities in the tumor microenvironment (TME) are important factors that induce tumor metabolic reprogramming (41, 42). The TME is a complex network composed of the extracellular matrix, hematopoietic cells, and mesenchymal cells. An aberrant tumor immune microenvironment is conducive to the differentiation of tumor cells into highly aggressive cell subtypes and suppression of antitumor immune responses, leading to tumor progression (43). In this study, we further explored the relationship between tryptophan metabolism and the tumor immune microenvironment in BLCA. The CIBERSORT algorithm demonstrated that five immune cells (CD8+ T cells, macrophages M1 and M2, regulatory T cells (Tregs), and B cells) were differentially expressed between the two risk subgroups. The low-risk group was associated with a higher level of CD8+ T cell infiltration, indicating that the immune system in the low-risk group exerted a more effective response against tumor proliferation. Moreover, the infiltration of macrophages M1 was negatively associated with risk scores, whereas macrophage M2 was positively associated with risk scores. Macrophages M1 inhibit tumor growth by directly killing tumor cells or stimulating other immune cells to enhance the host antitumor immune response, whereas macrophages M2 suppress immune system activity, thereby weakening antitumor immunity. IPS analysis revealed that patients in the low-risk groups had better immunotherapy efficacy and were more likely to benefit from immunotherapy. These results indicate that differences in tryptophan metabolism between the high- and low-risk groups could affect the TME. The high-risk group, which represents higher tryptophan metabolism, is more likely to form an immunosuppressive microenvironment, induce immune escape of tumor cells, and result in a worse immunotherapy response and prognosis.

In conclusion, the tryptophan metabolism- and immune-related gene risk model can effectively predict patient prognosis and immunotherapy response and is an effective prognostic model for BLCA. Our study further explored the potential applications of tryptophan metabolism in improving the response to immunotherapy and has crucial implications for individualized therapy to improve the prognosis of patients with BLCA. However, further validation using basic experiments is required to confirm our findings.

## Data availability statement

The datasets presented in this study can be found in online repositories. The names of the repository/repositories and accession number(s) can be found in the article/**Supplementary Material**.

## Ethics statement

The studies involving humans were approved by Institutional Review Board of Qilu Hospital. The studies were conducted in accordance with the local legislation and institutional requirements. The participants provided their written informed consent to participate in this study.

## Author contributions

LX: Formal Analysis, Investigation, Methodology, Software, Validation, Writing – review & editing. GQ: Data curation, Resources, Writing – original draft. ZZ: Conceptualization, Project administration, Resources, Software, Writing – original draft. HZ: Data curation, Resources, Validation, Writing – review & editing. GZ: Funding acquisition, Project administration, Supervision, Validation, Visualization, Writing – original draft.
