# Supplementary figures and images for: Identification of tryptophan metabolism- and immune-related genes signature and prediction of immune infiltration landscape in bladder urothelial carcinoma
Source: Front Immunol. 2023 Oct 26;14:1283792. doi: 10.3389/fimmu.2023.1283792 (PMC10637370; doi:10.3389/fimmu.2023.1283792)

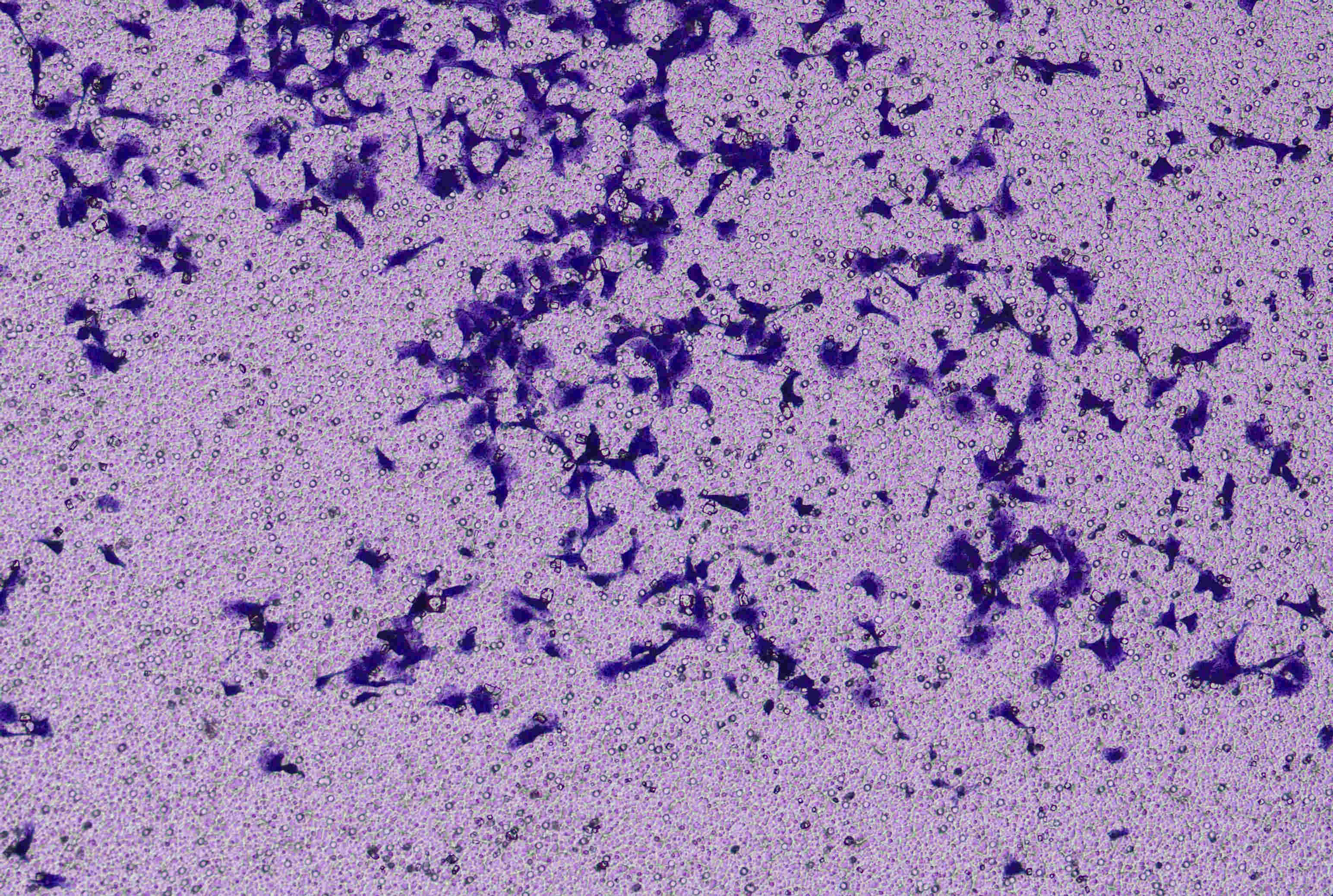

Supplement: Supplementary file 1 [file DataSheet_1.zip › Raw Data/Transwell/5637/INV-1-112.jpg]

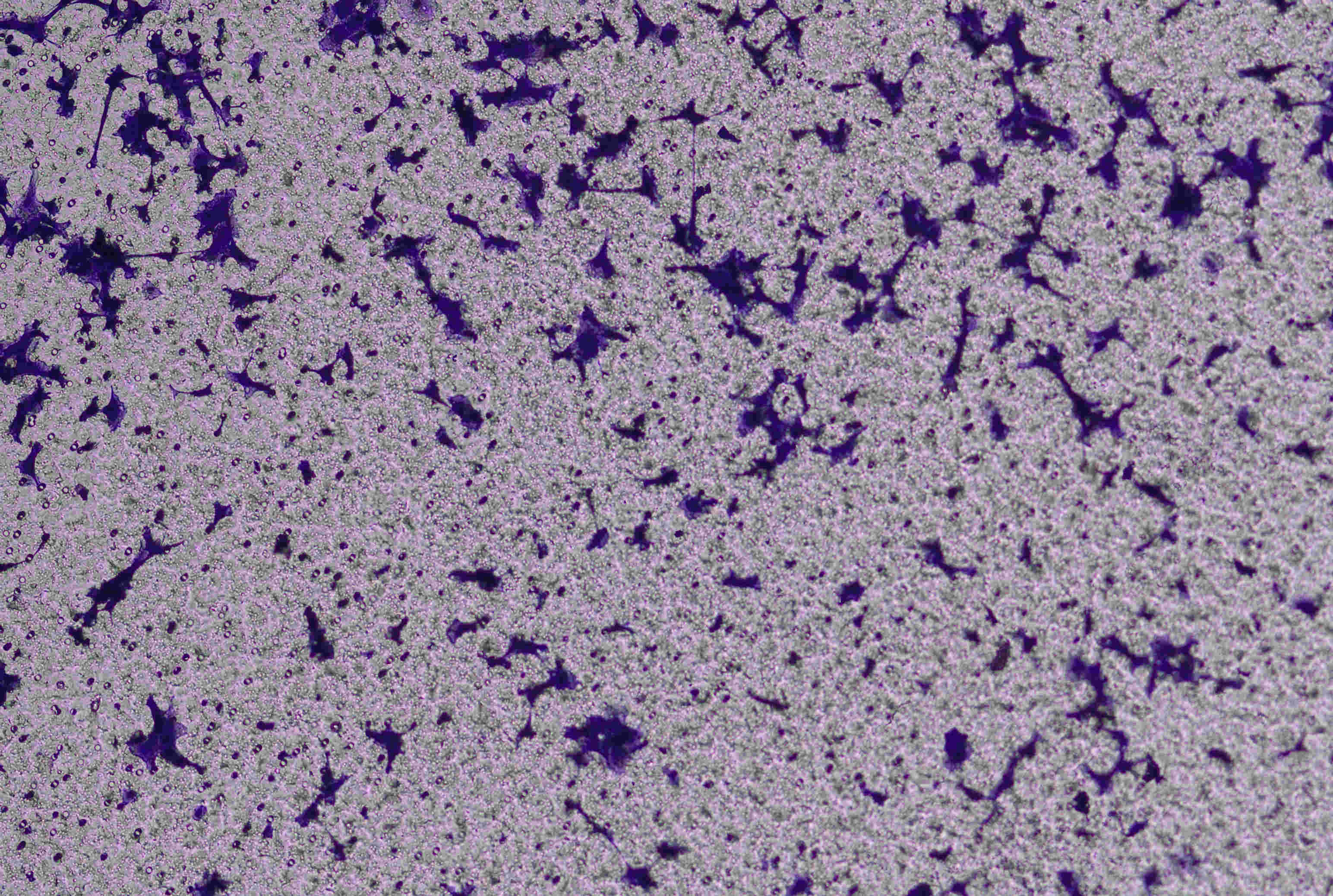

Supplement: Supplementary file 1 [file DataSheet_1.zip › Raw Data/Transwell/5637/INV-1-113.jpg]

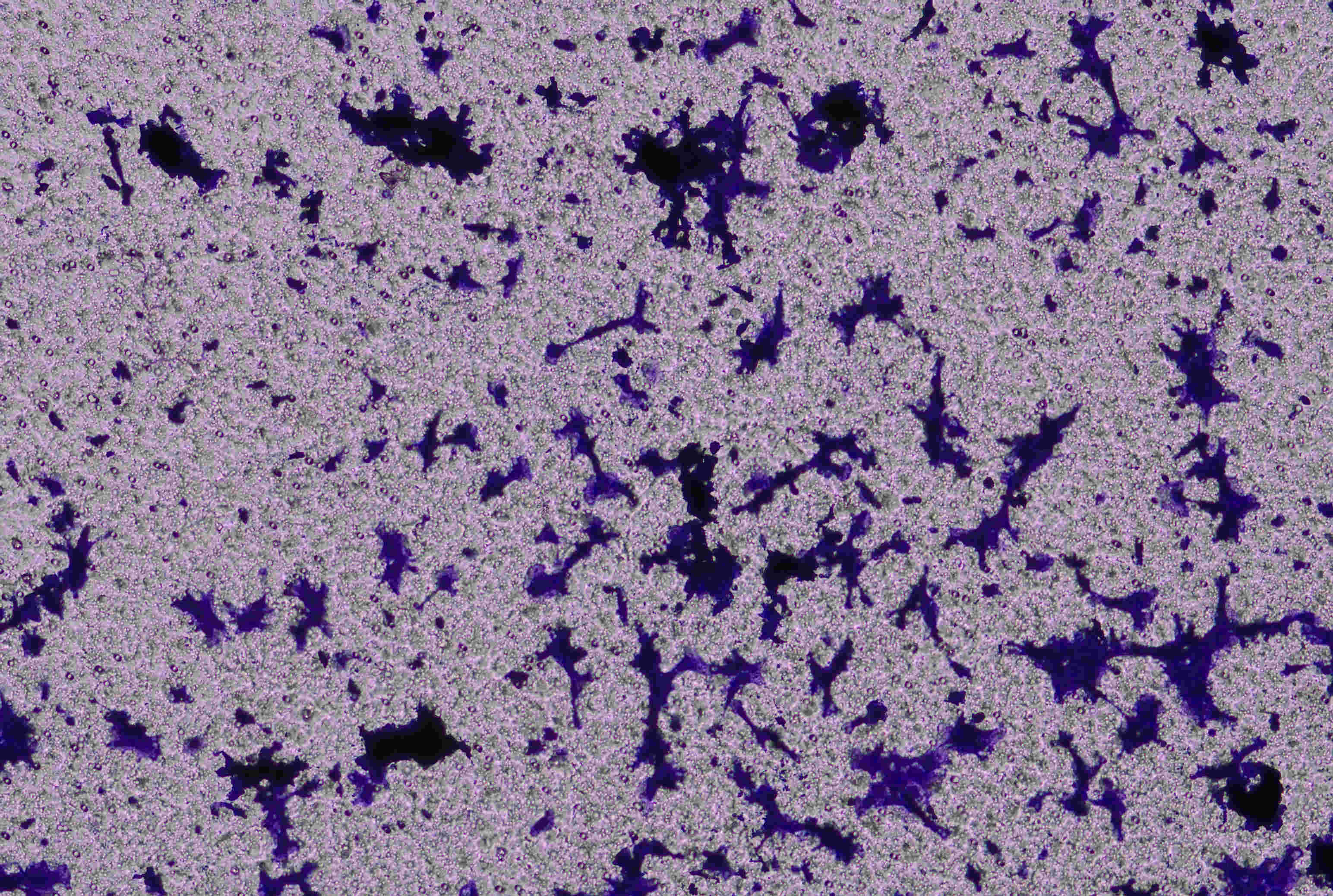

Supplement: Supplementary file 1 [file DataSheet_1.zip › Raw Data/Transwell/5637/INV-1-124.jpg]

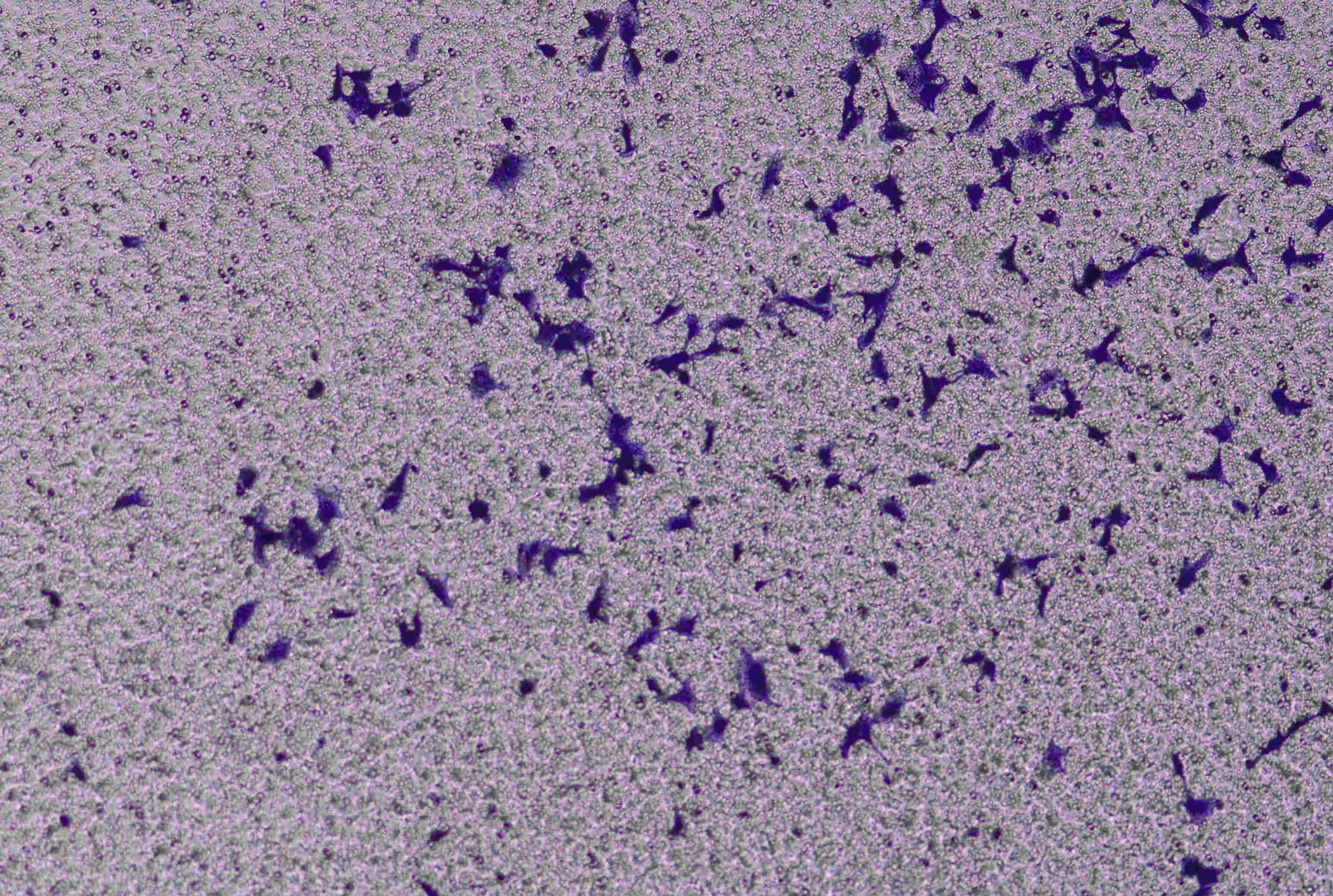

Supplement: Supplementary file 1 [file DataSheet_1.zip › Raw Data/Transwell/5637/INV-2-108.jpg]

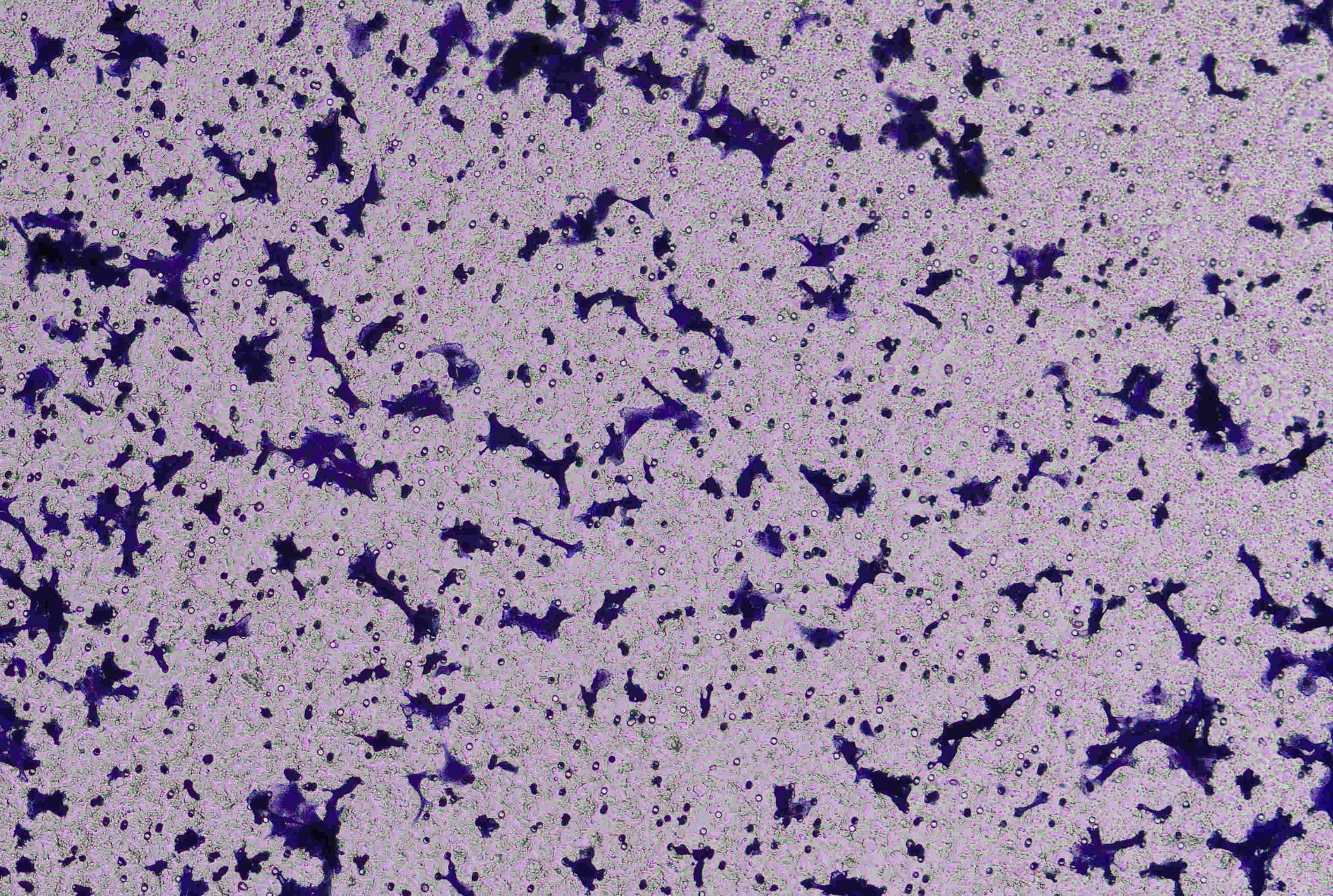

Supplement: Supplementary file 1 [file DataSheet_1.zip › Raw Data/Transwell/5637/INV-2-121.jpg]

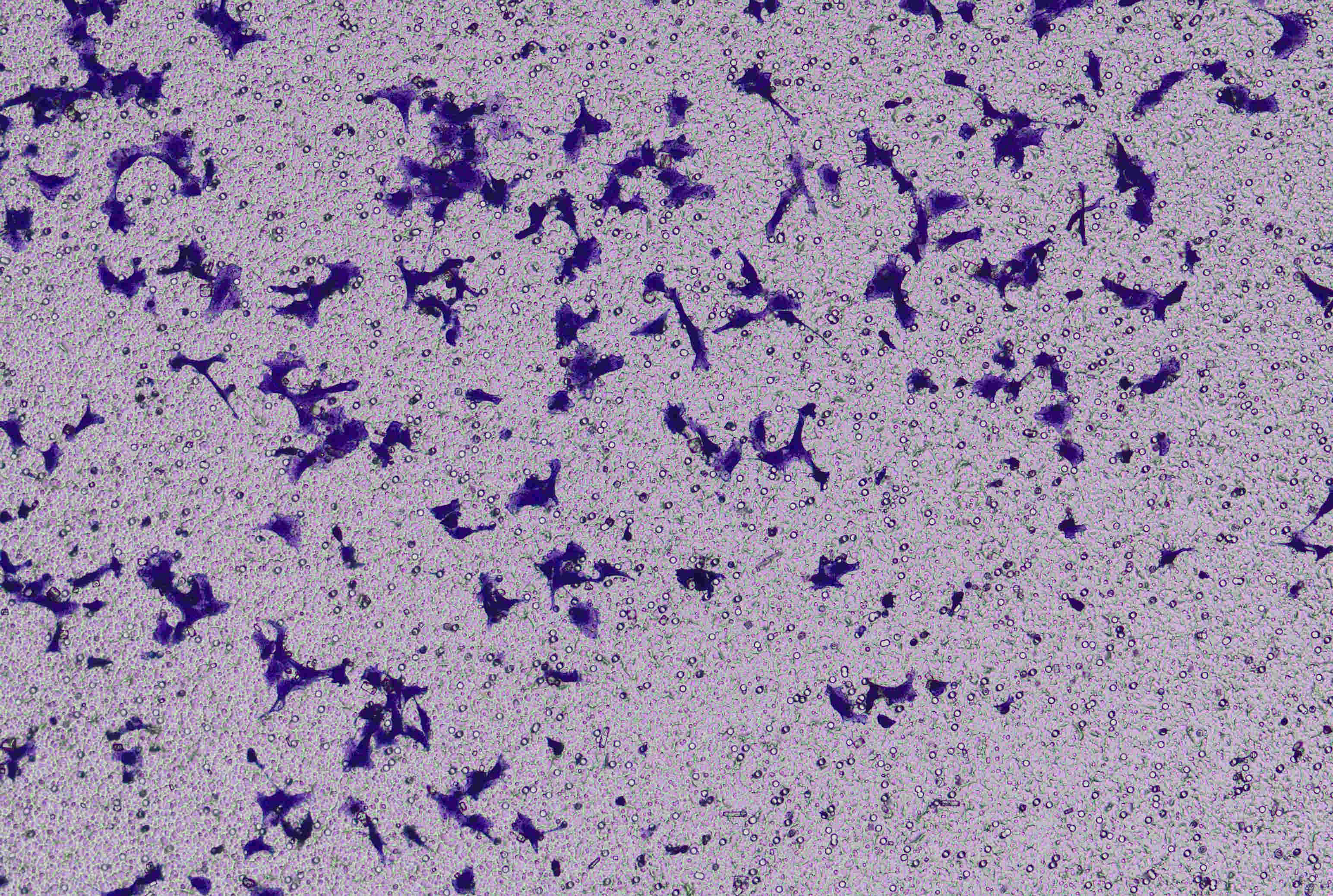

Supplement: Supplementary file 1 [file DataSheet_1.zip › Raw Data/Transwell/5637/INV-2-97.jpg]

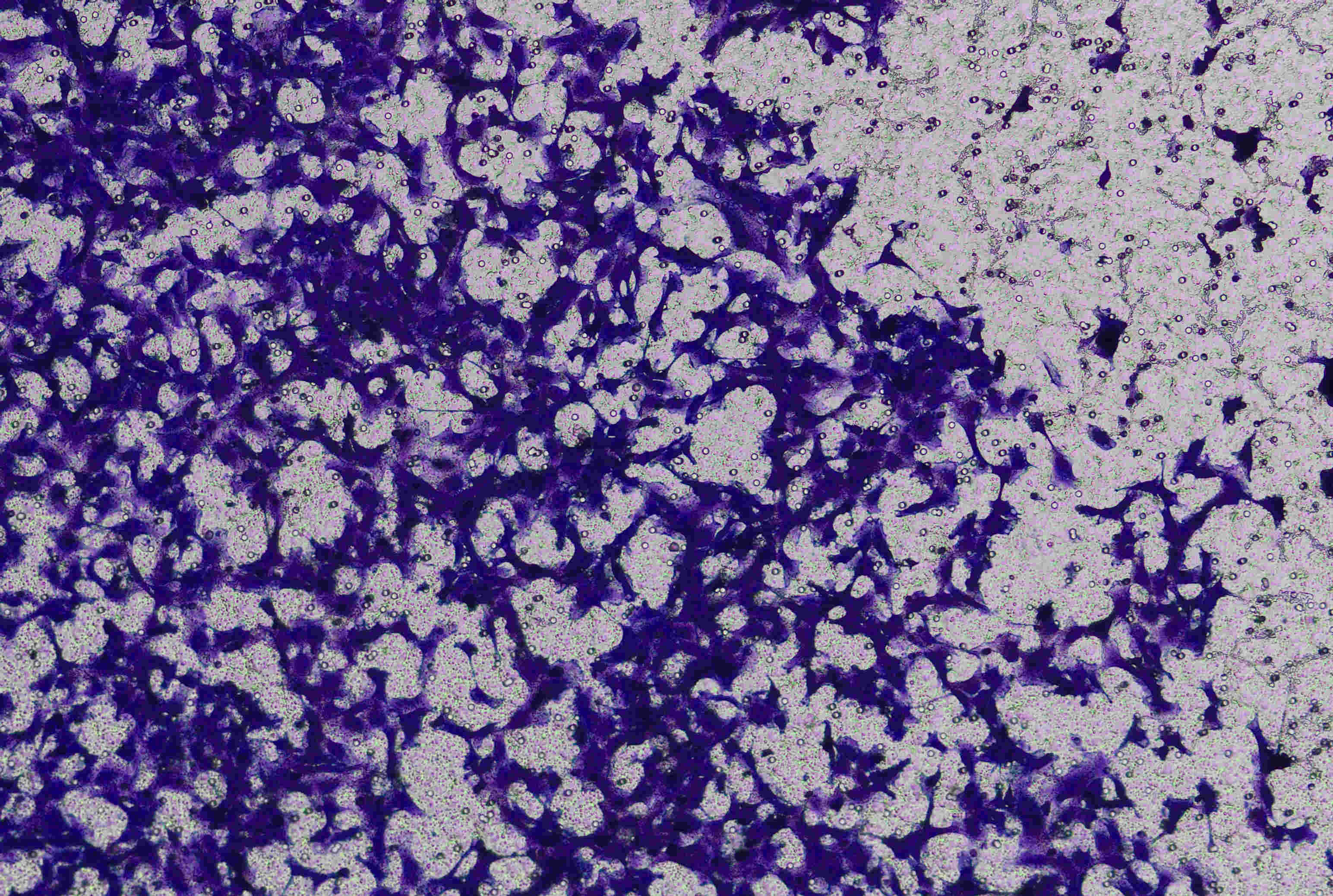

Supplement: Supplementary file 1 [file DataSheet_1.zip › Raw Data/Transwell/5637/INV-NC-160.jpg]

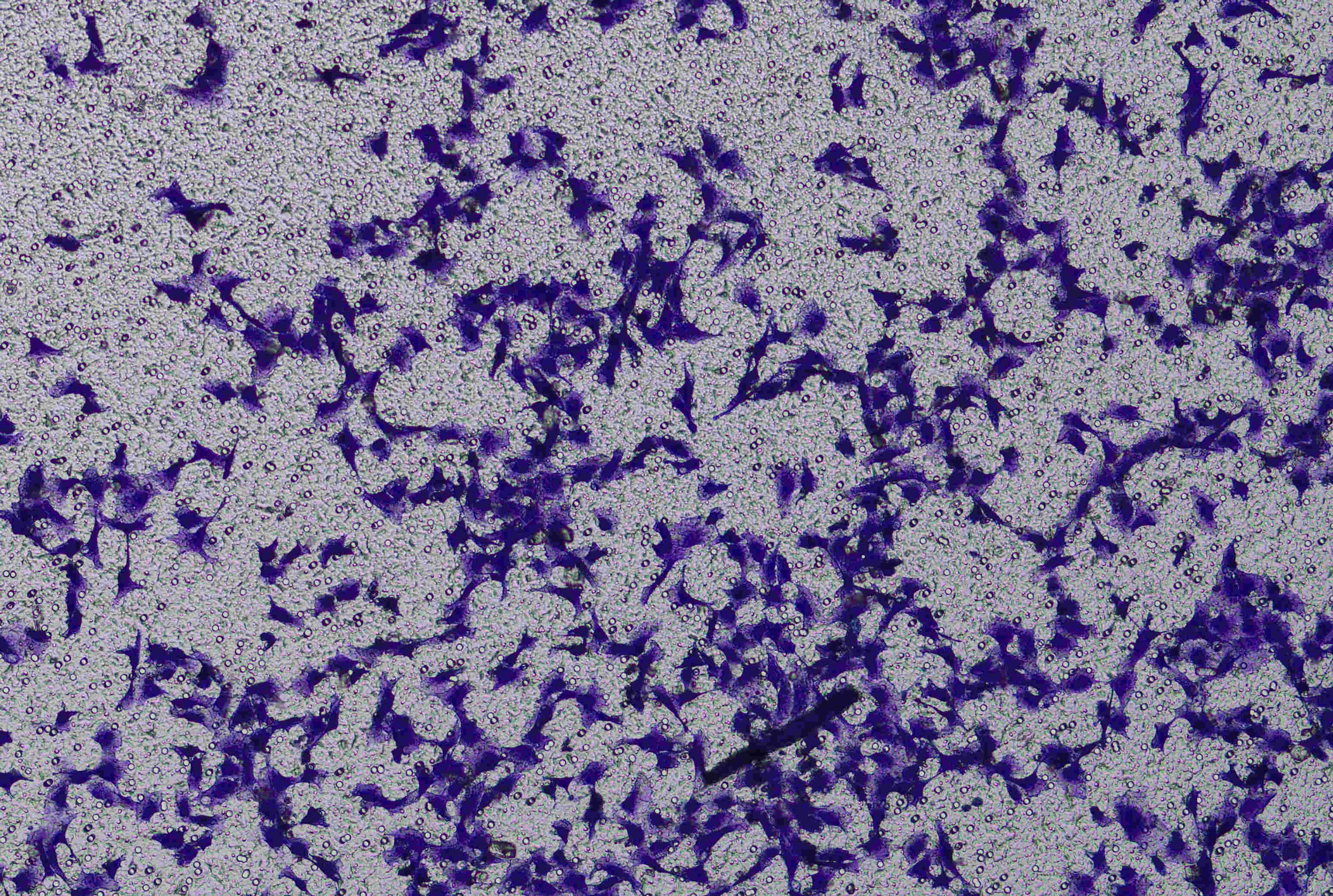

Supplement: Supplementary file 1 [file DataSheet_1.zip › Raw Data/Transwell/5637/INV-NC-166.jpg]

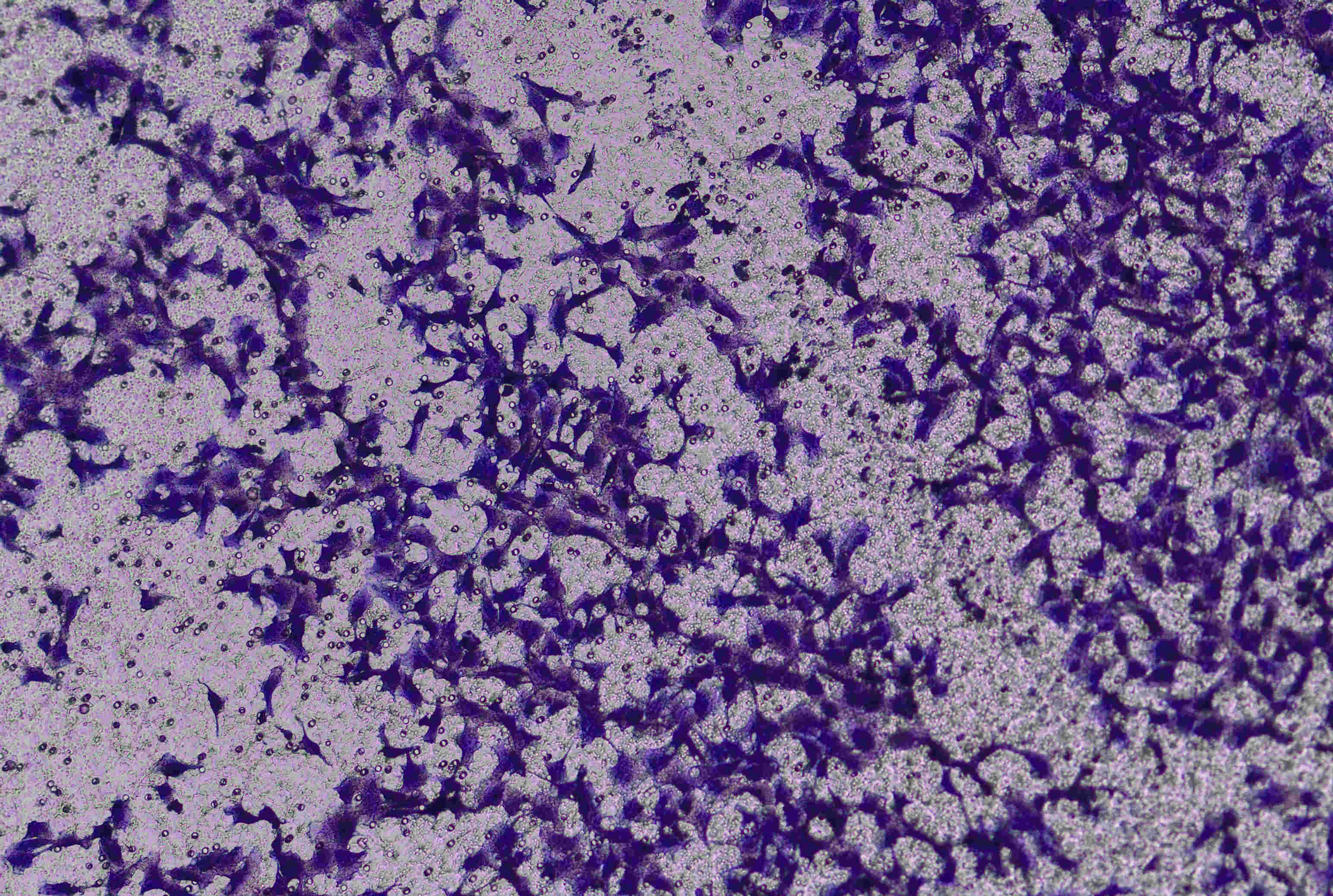

Supplement: Supplementary file 1 [file DataSheet_1.zip › Raw Data/Transwell/5637/INV-NC-178.jpg]

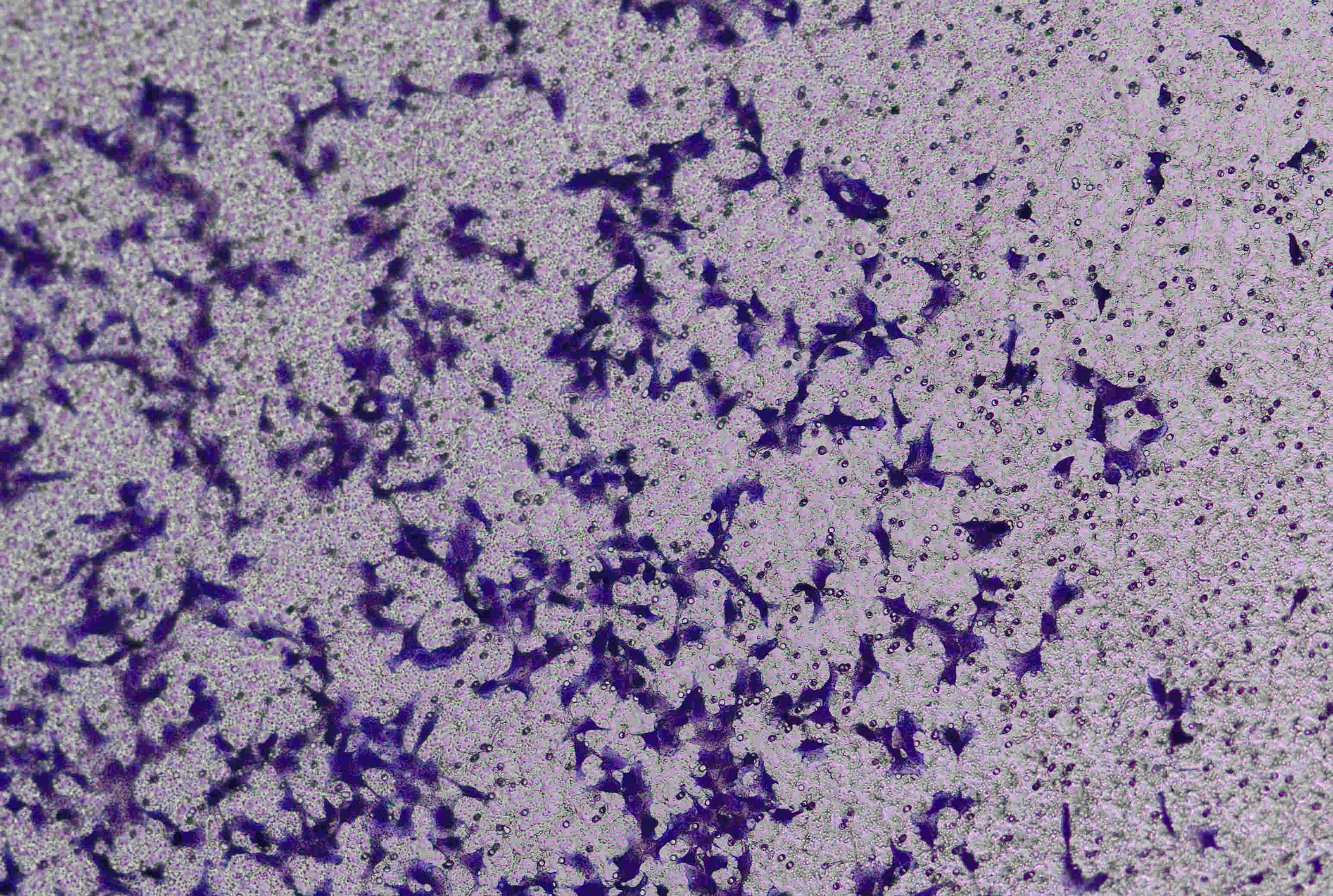

Supplement: Supplementary file 1 [file DataSheet_1.zip › Raw Data/Transwell/5637/MIG-1-128.jpg]

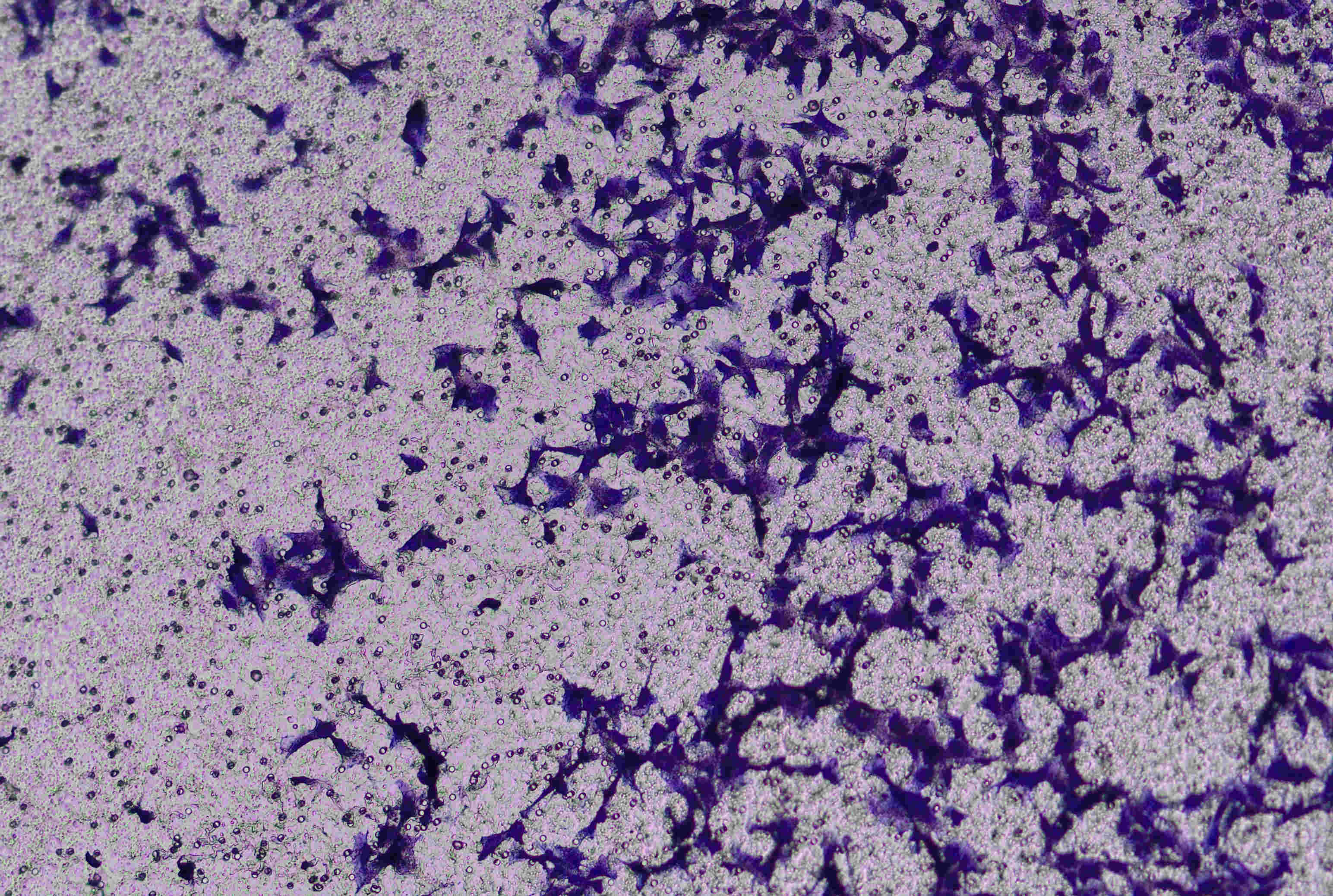

Supplement: Supplementary file 1 [file DataSheet_1.zip › Raw Data/Transwell/5637/MIG-1-135.jpg]

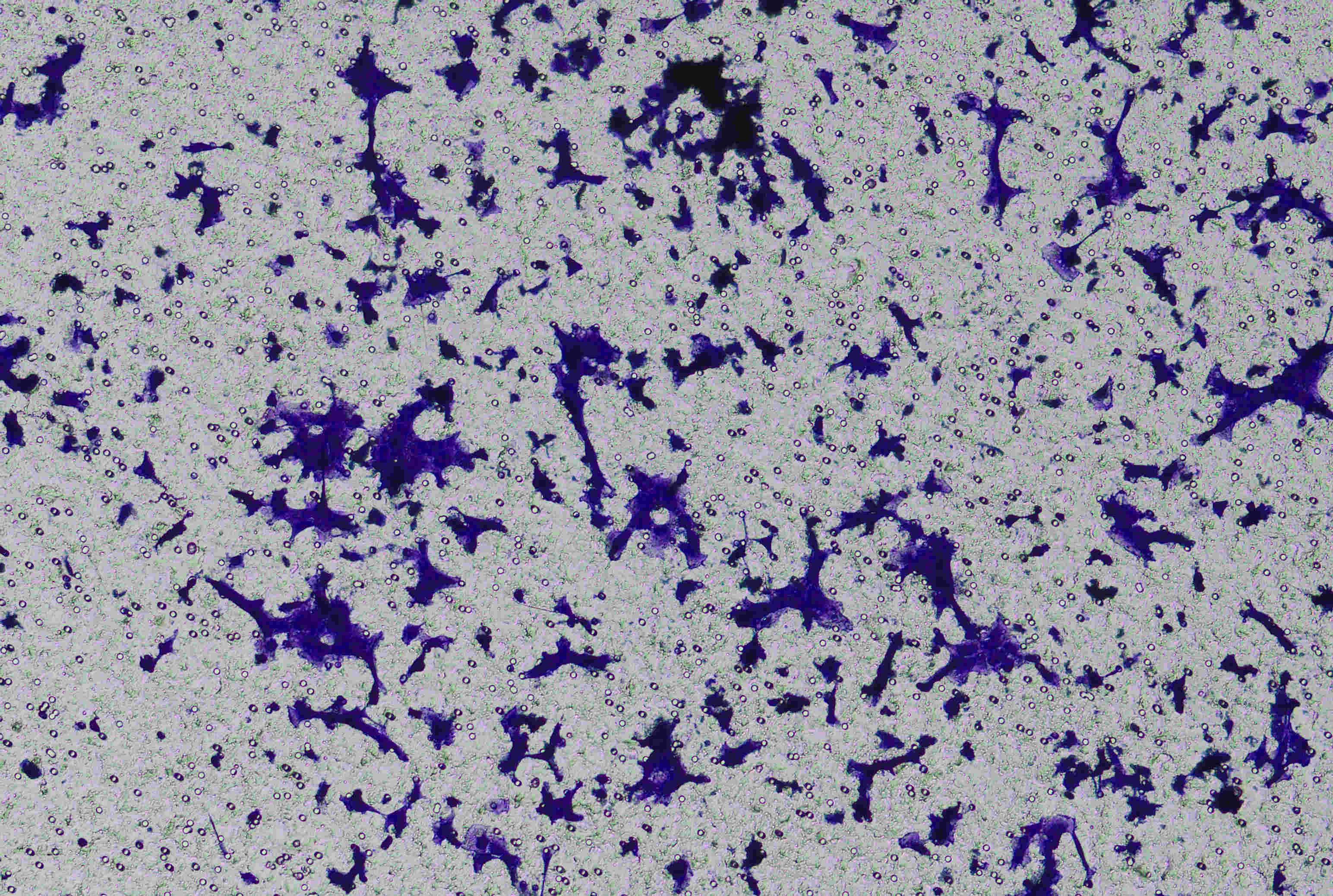

Supplement: Supplementary file 1 [file DataSheet_1.zip › Raw Data/Transwell/5637/MIG-1-143.jpg]

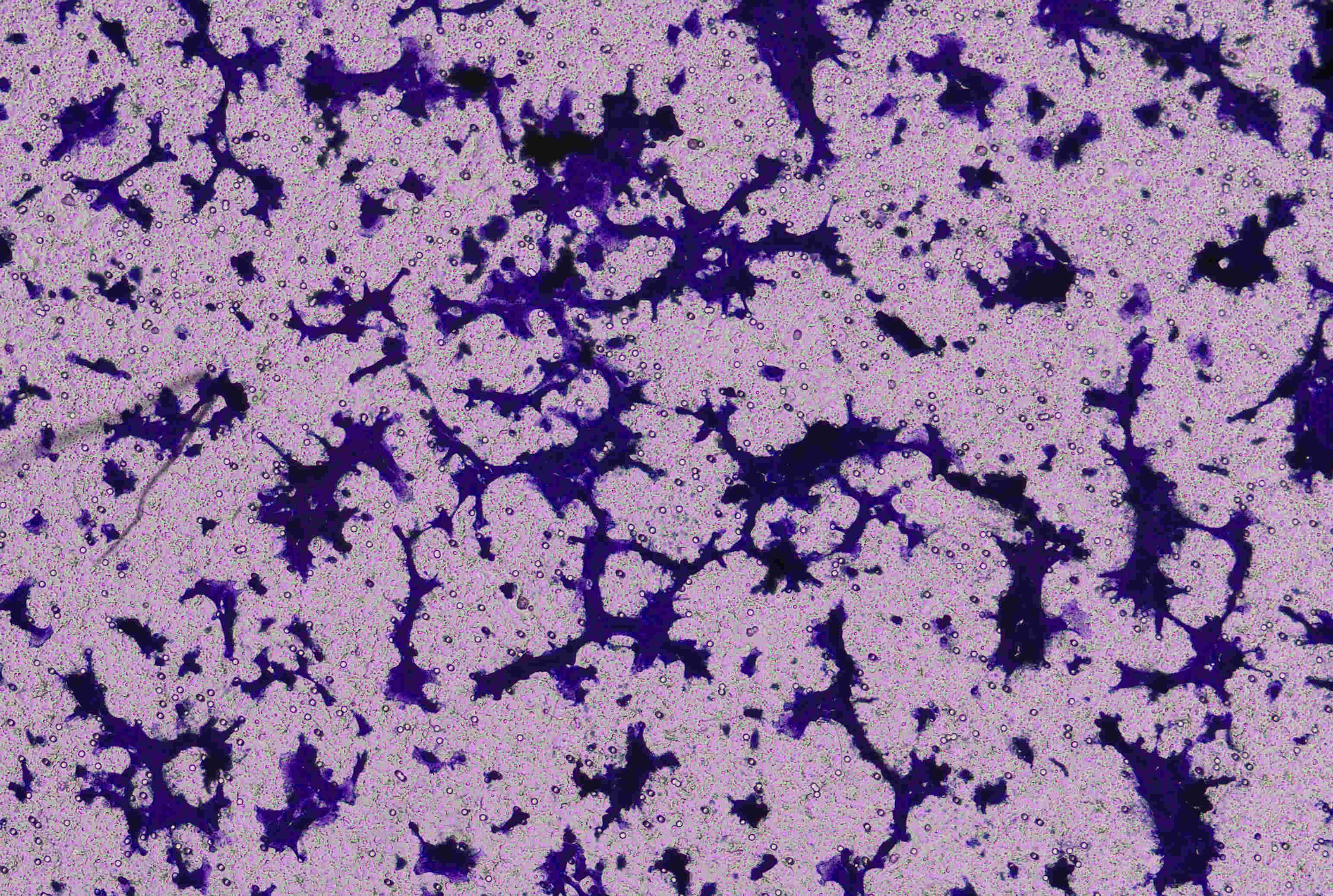

Supplement: Supplementary file 1 [file DataSheet_1.zip › Raw Data/Transwell/5637/MIG-2-139.jpg]

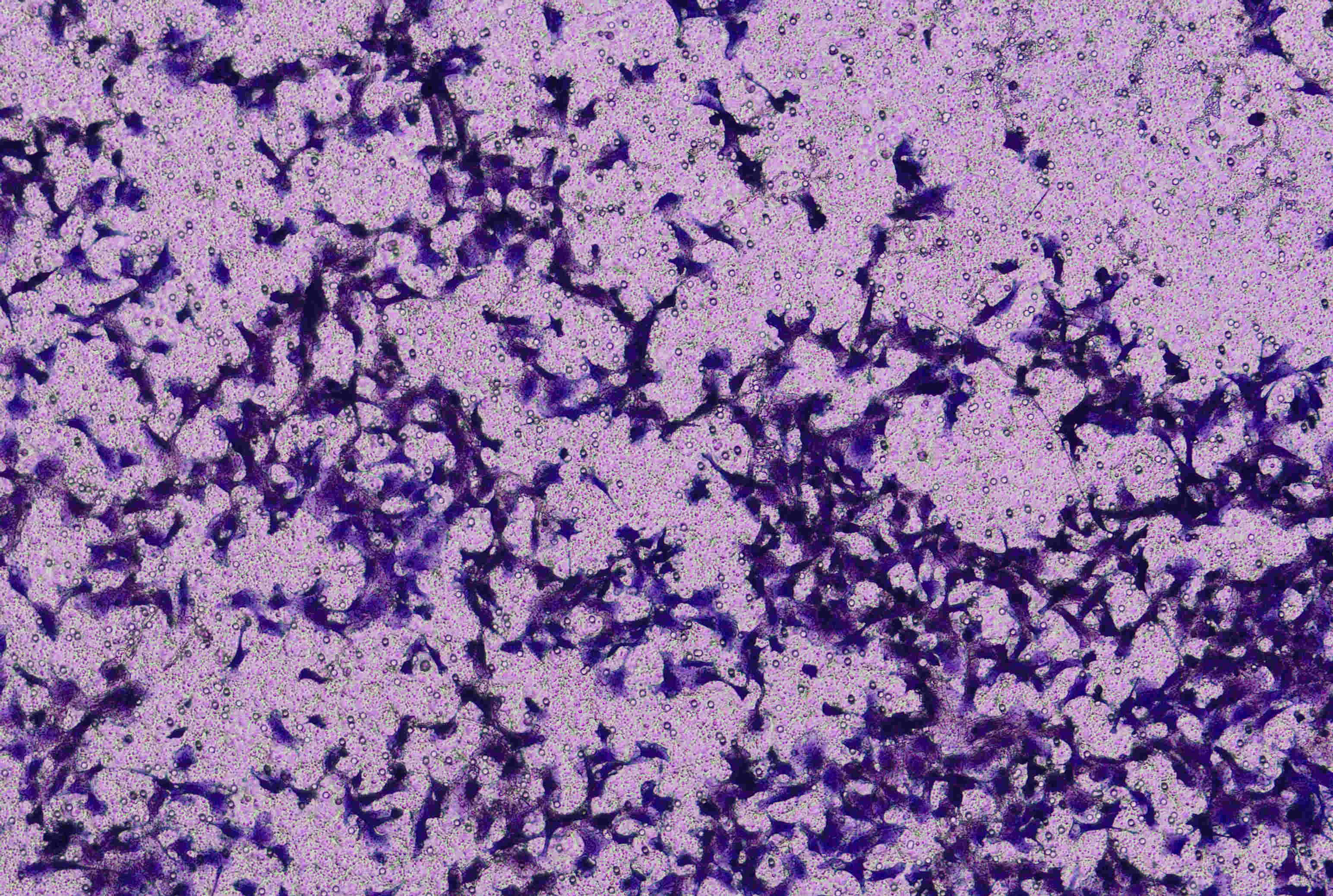

Supplement: Supplementary file 1 [file DataSheet_1.zip › Raw Data/Transwell/5637/MIG-2-152.jpg]

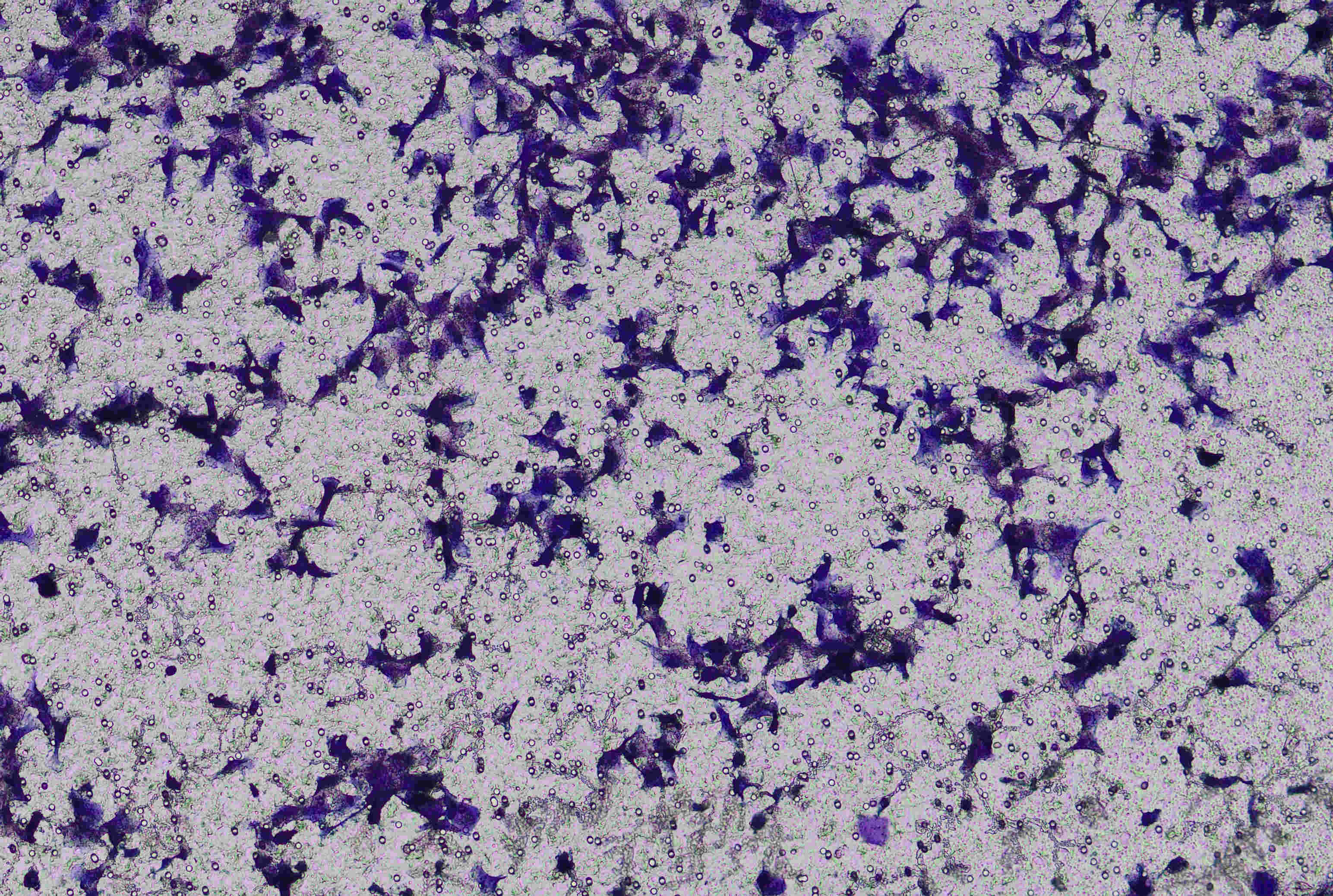

Supplement: Supplementary file 1 [file DataSheet_1.zip › Raw Data/Transwell/5637/MIG-2-155.jpg]

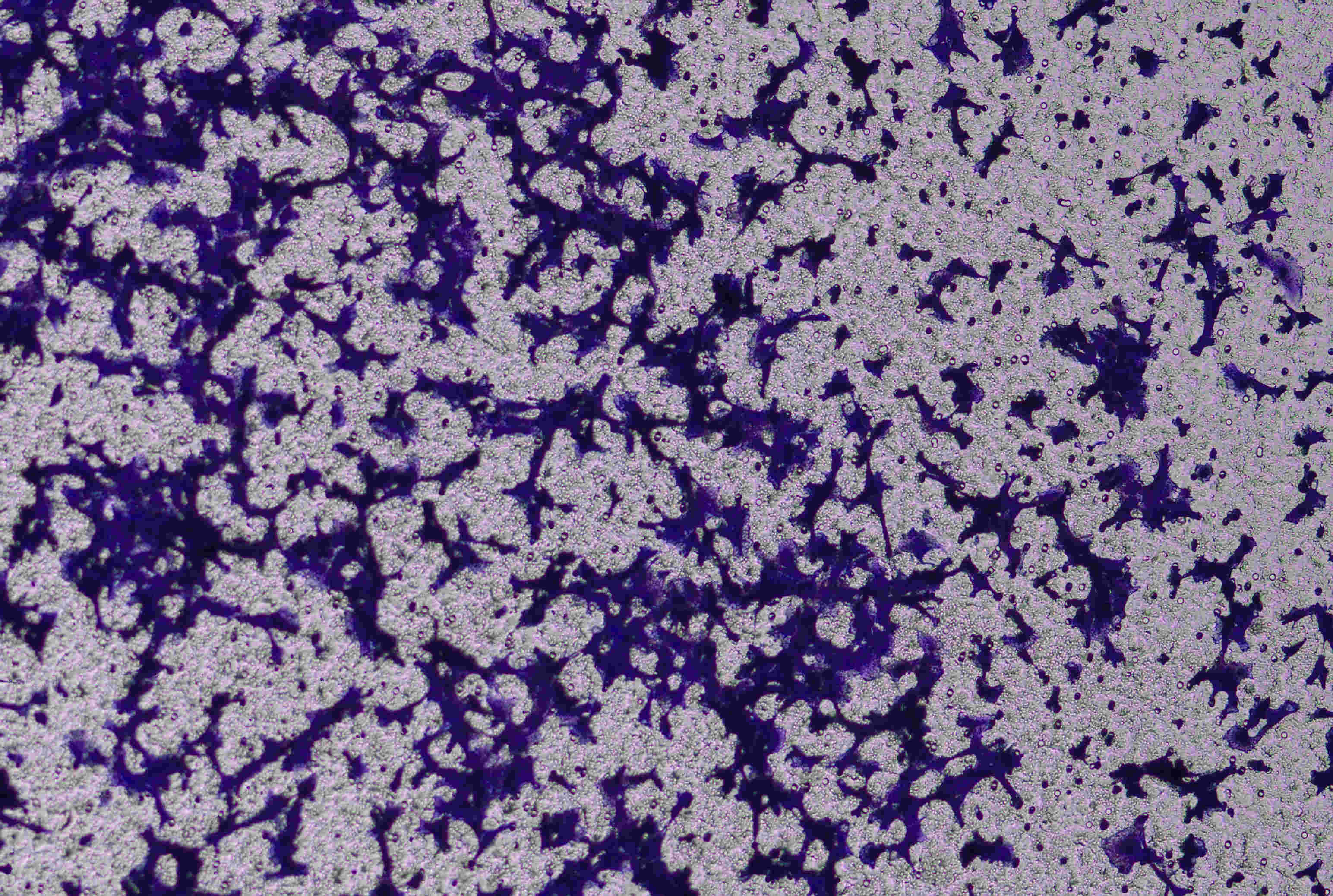

Supplement: Supplementary file 1 [file DataSheet_1.zip › Raw Data/Transwell/5637/MIG-NC-182.jpg]

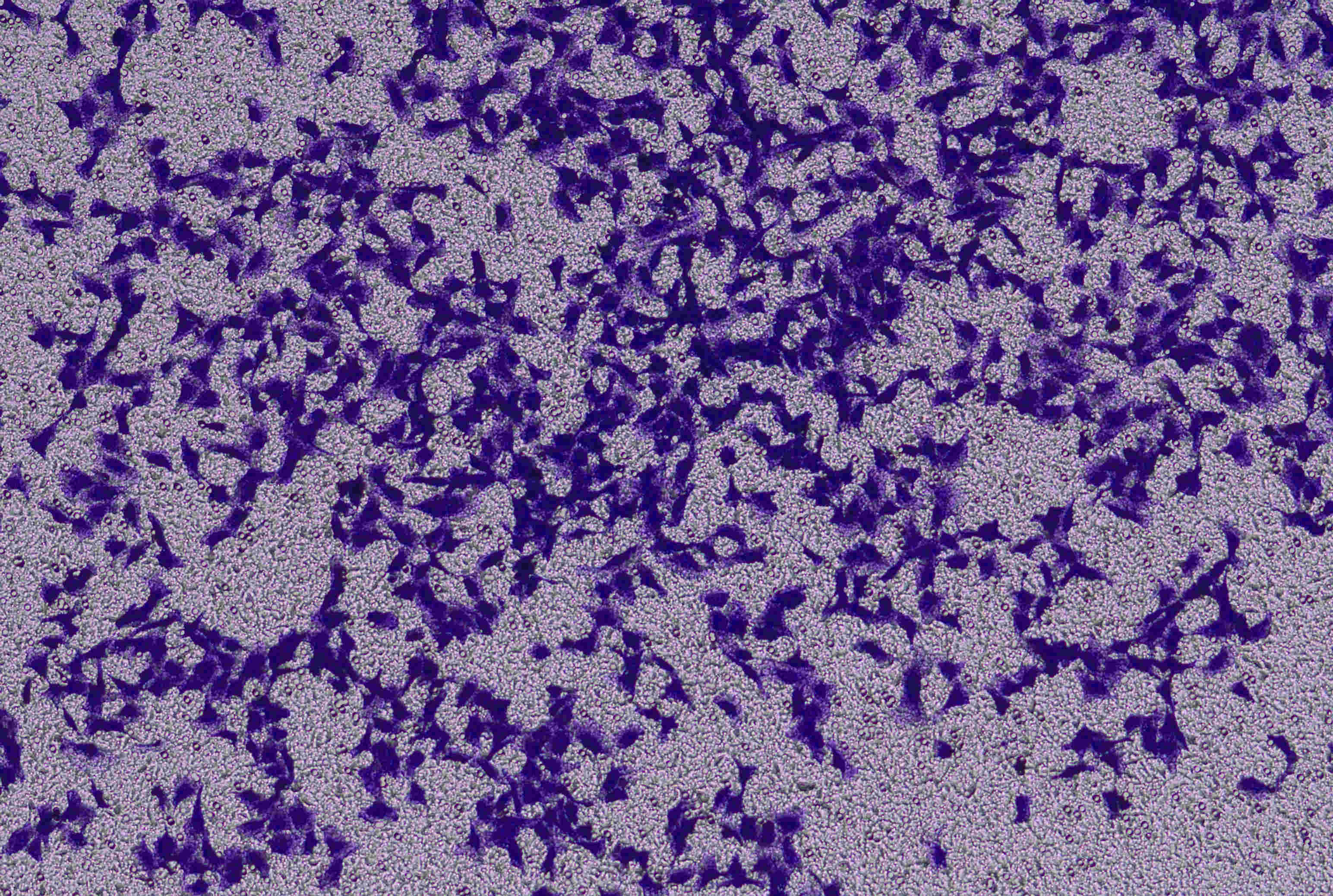

Supplement: Supplementary file 1 [file DataSheet_1.zip › Raw Data/Transwell/5637/MIG-NC-198.jpg]

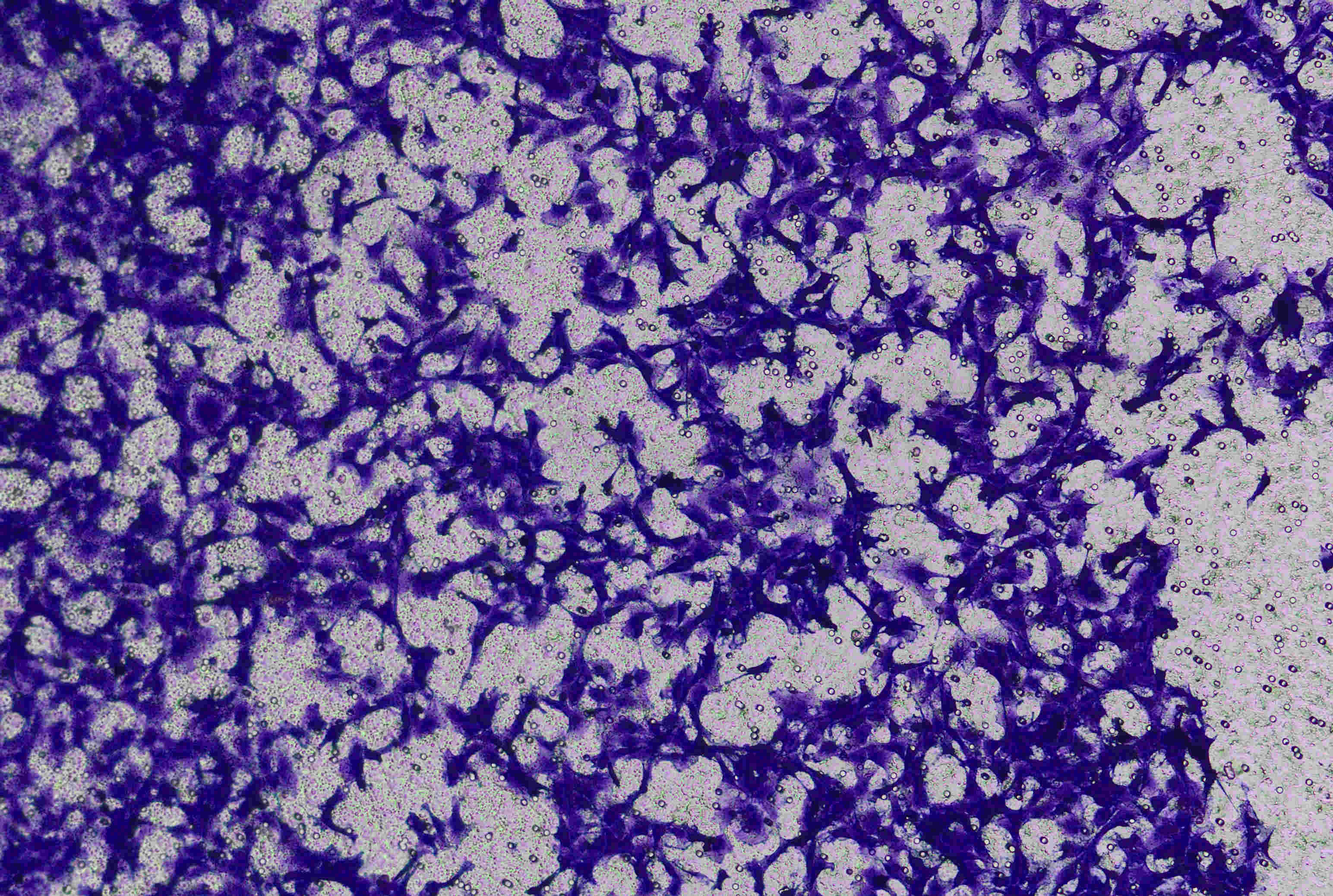

Supplement: Supplementary file 1 [file DataSheet_1.zip › Raw Data/Transwell/5637/MIG-NC-212.jpg]

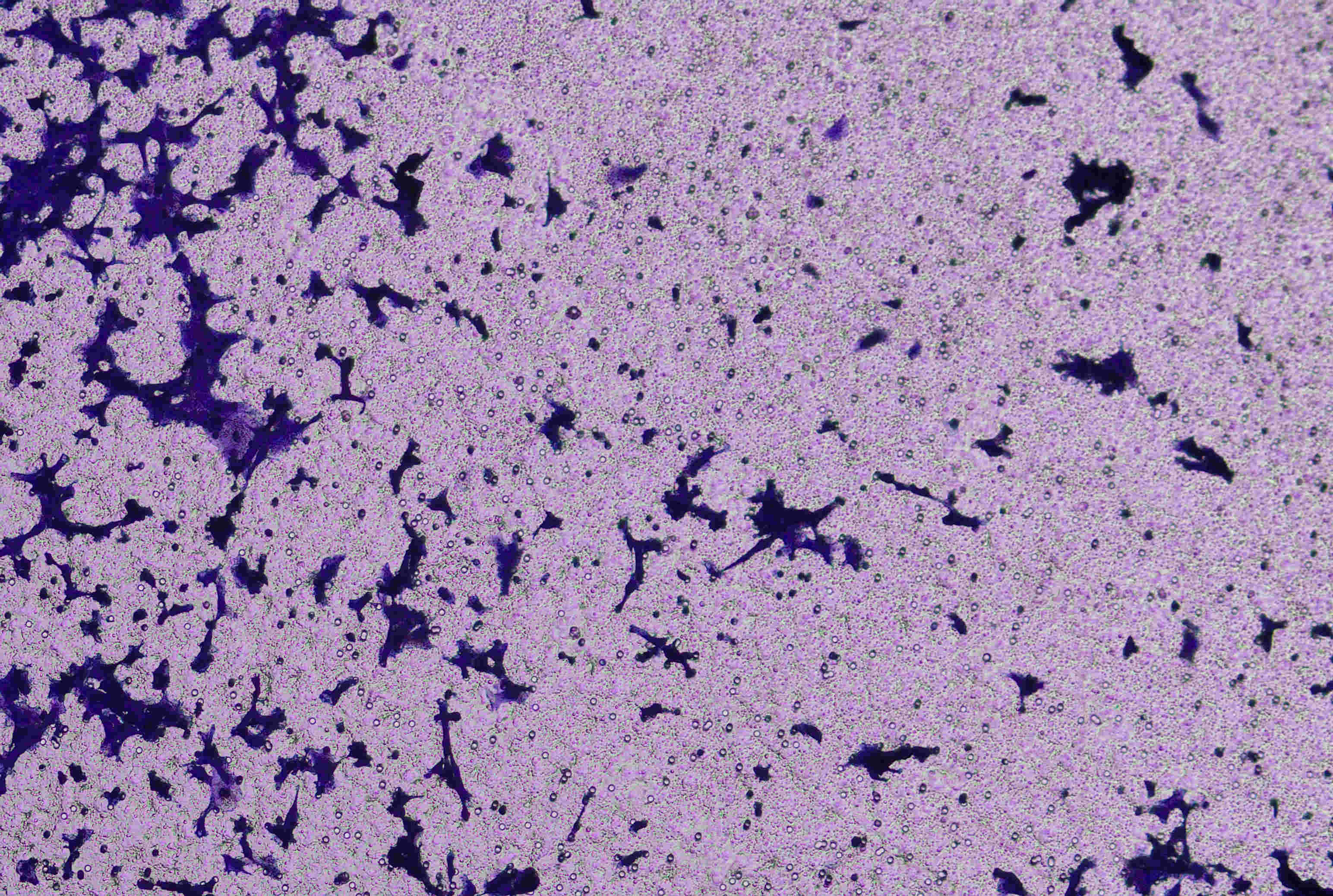

Supplement: Supplementary file 1 [file DataSheet_1.zip › Raw Data/Transwell/T24/INV-1-101.jpg]

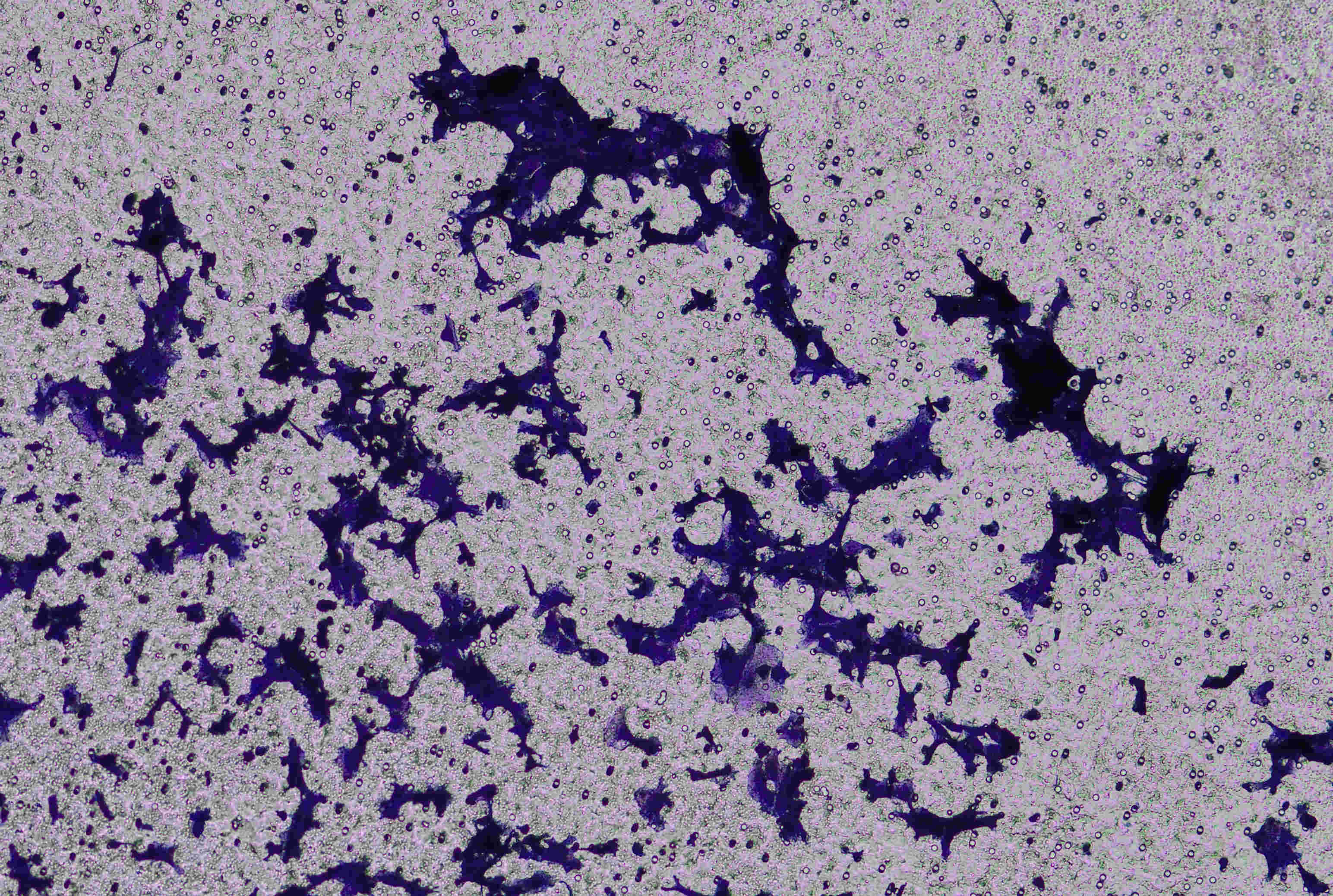

Supplement: Supplementary file 1 [file DataSheet_1.zip › Raw Data/Transwell/T24/INV-1-105.jpg]

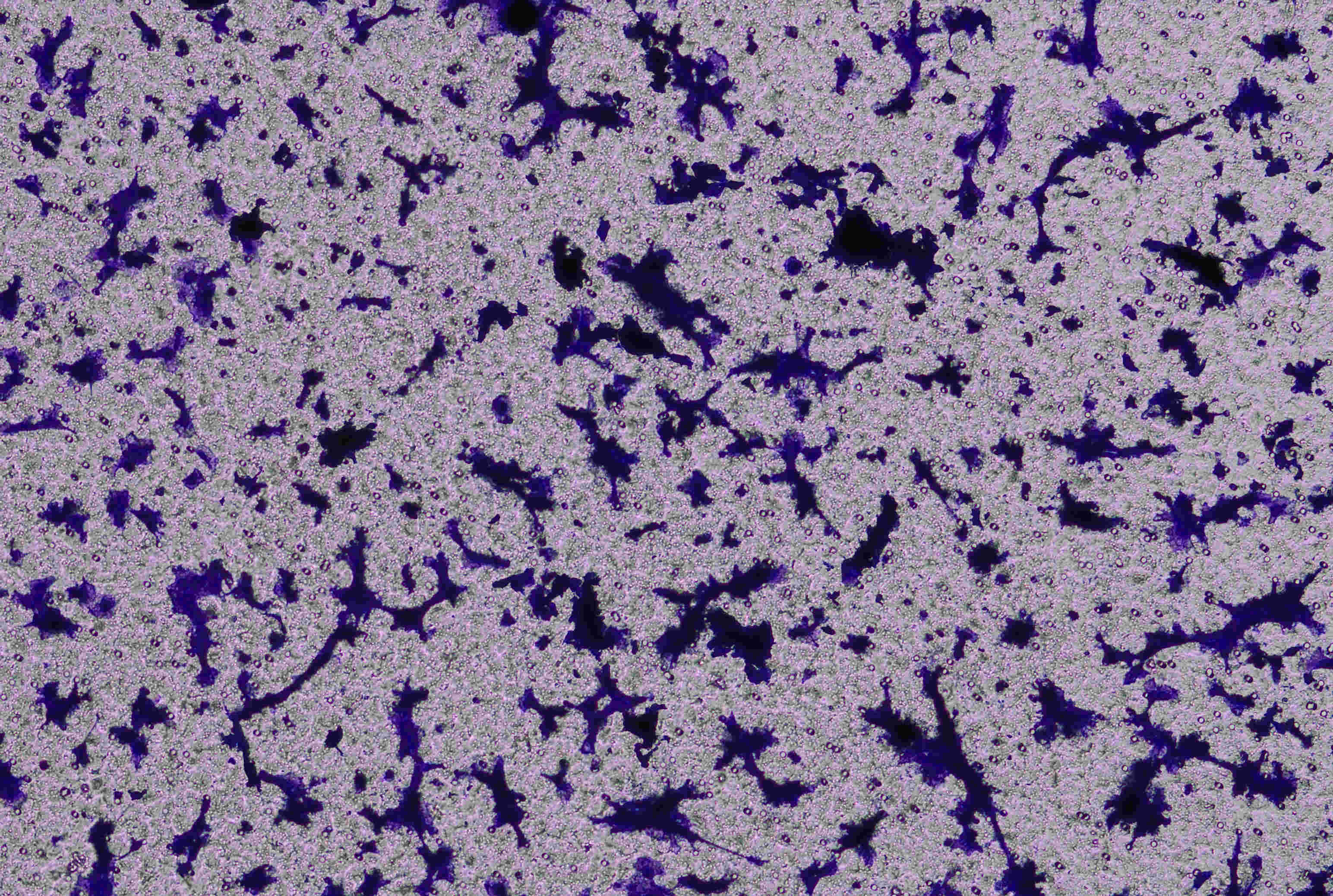

Supplement: Supplementary file 1 [file DataSheet_1.zip › Raw Data/Transwell/T24/INV-1-95.jpg]

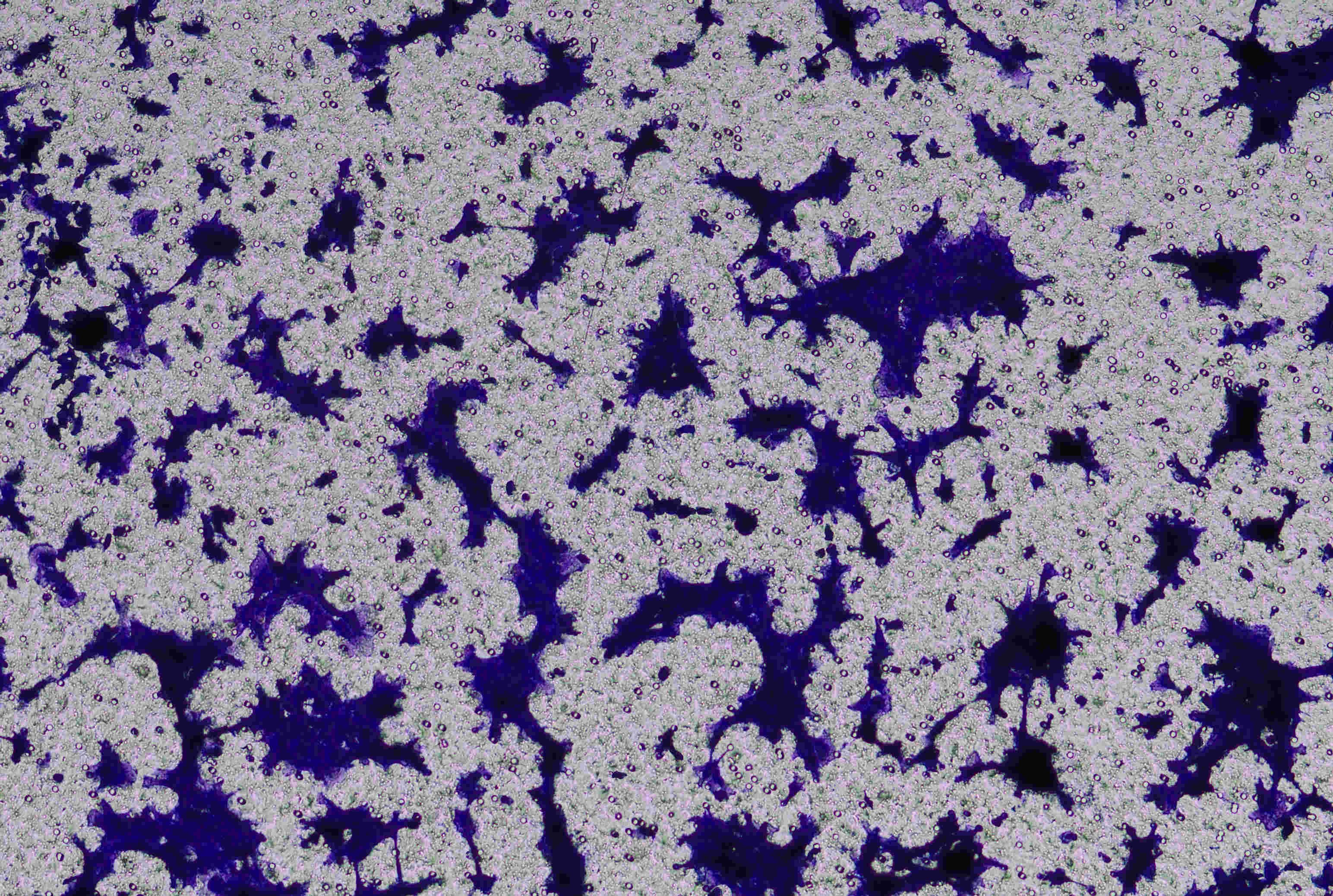

Supplement: Supplementary file 1 [file DataSheet_1.zip › Raw Data/Transwell/T24/INV-2-108.jpg]

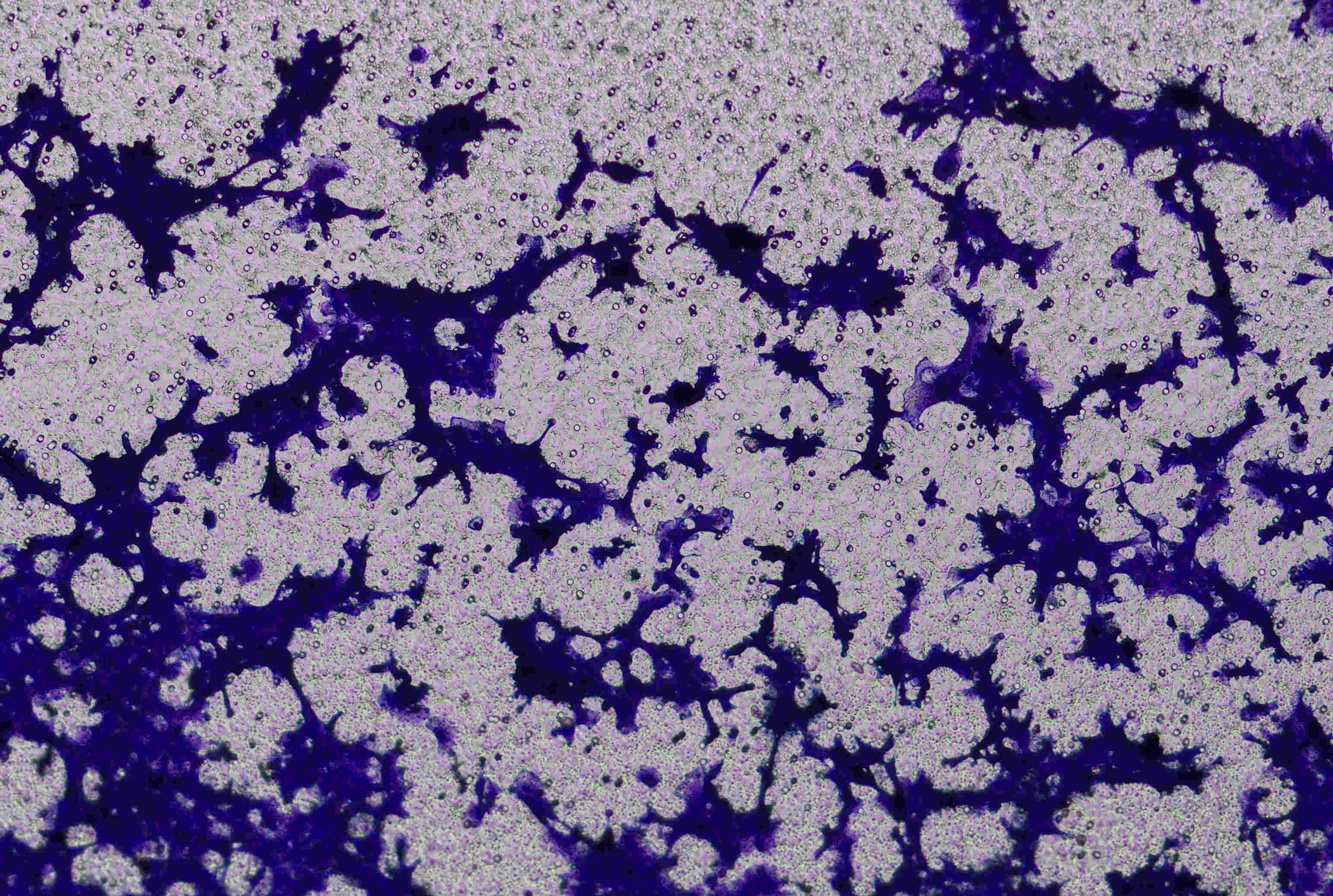

Supplement: Supplementary file 1 [file DataSheet_1.zip › Raw Data/Transwell/T24/INV-2-113.jpg]

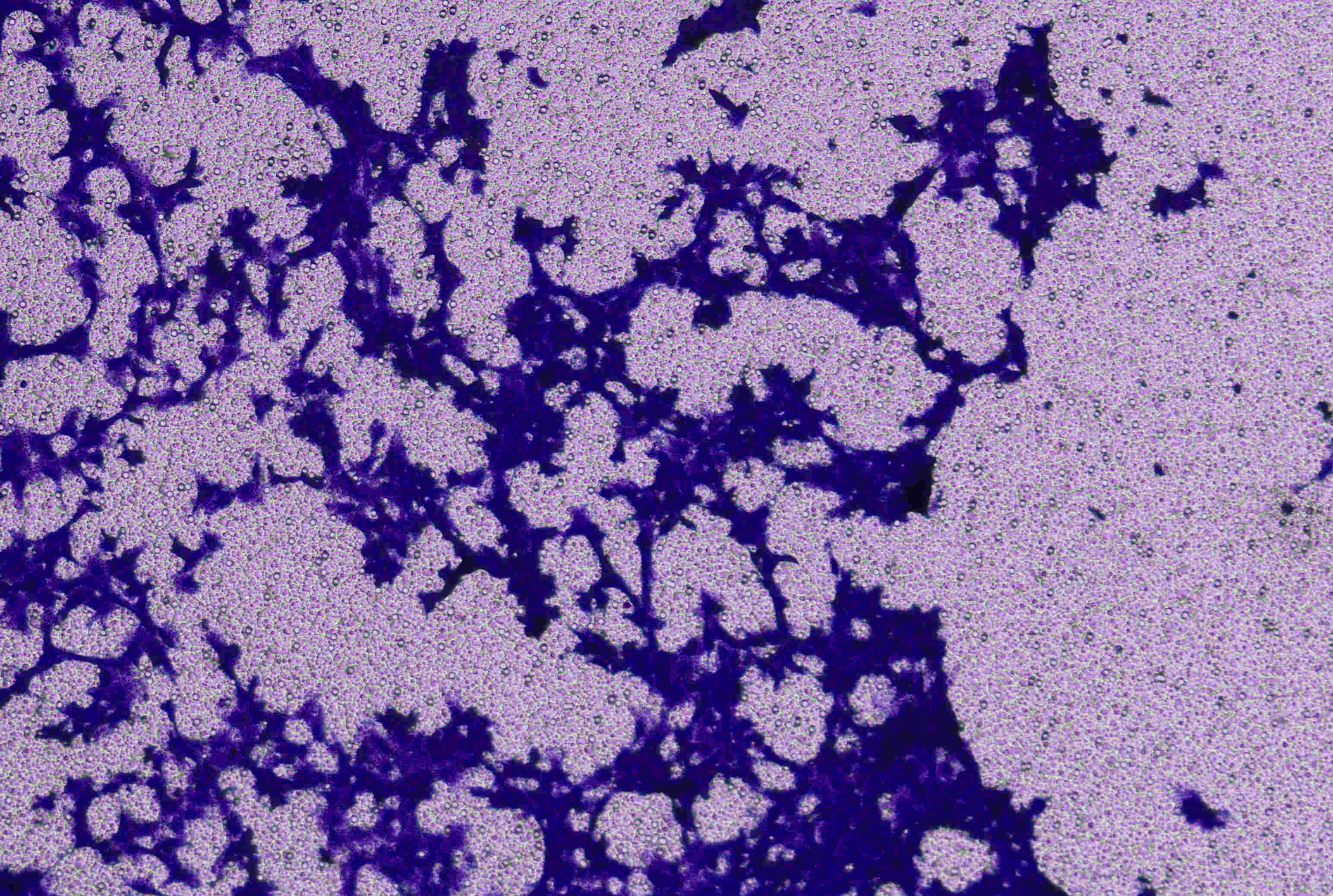

Supplement: Supplementary file 1 [file DataSheet_1.zip › Raw Data/Transwell/T24/INV-2-98.jpg]

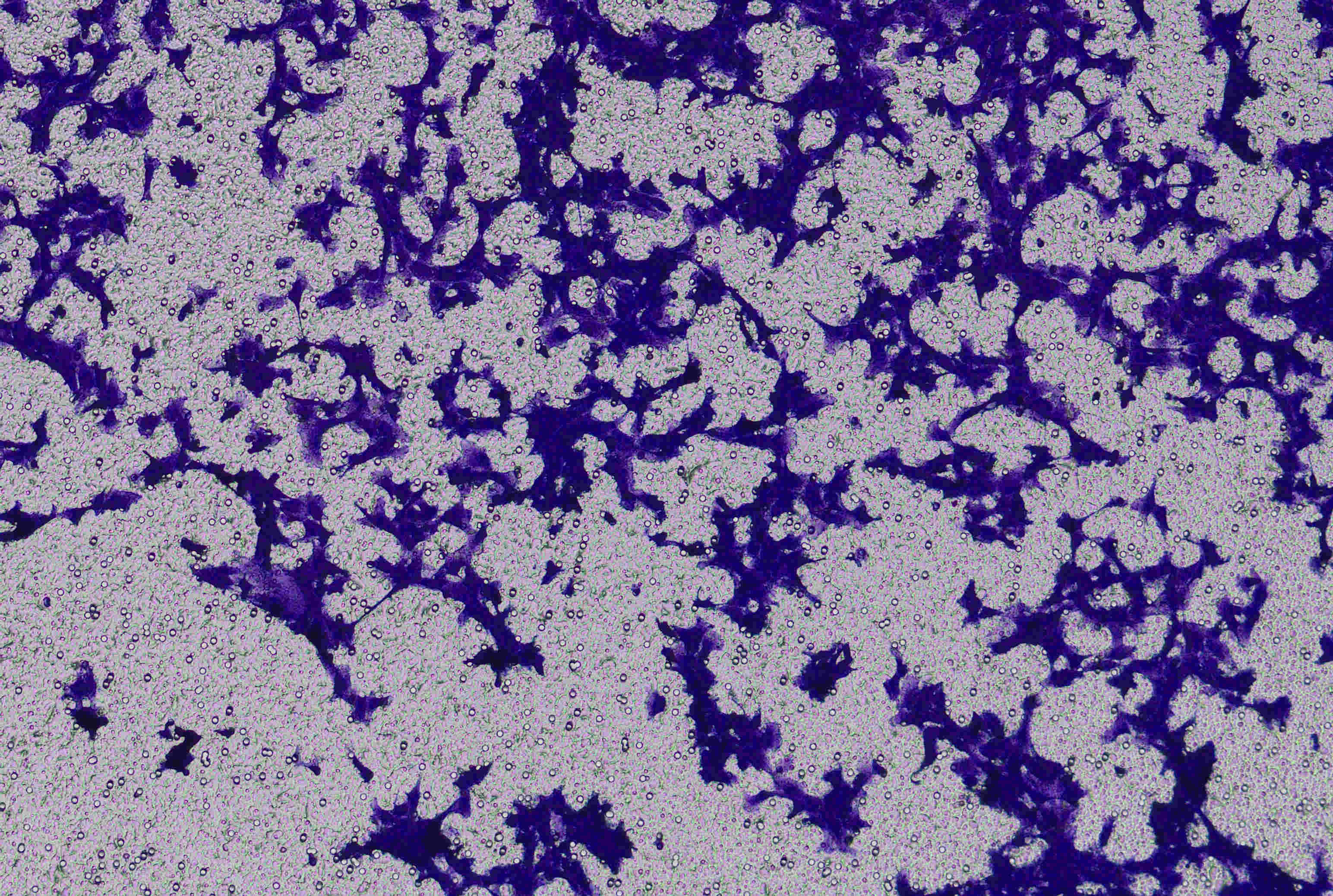

Supplement: Supplementary file 1 [file DataSheet_1.zip › Raw Data/Transwell/T24/INV-NC-128.jpg]

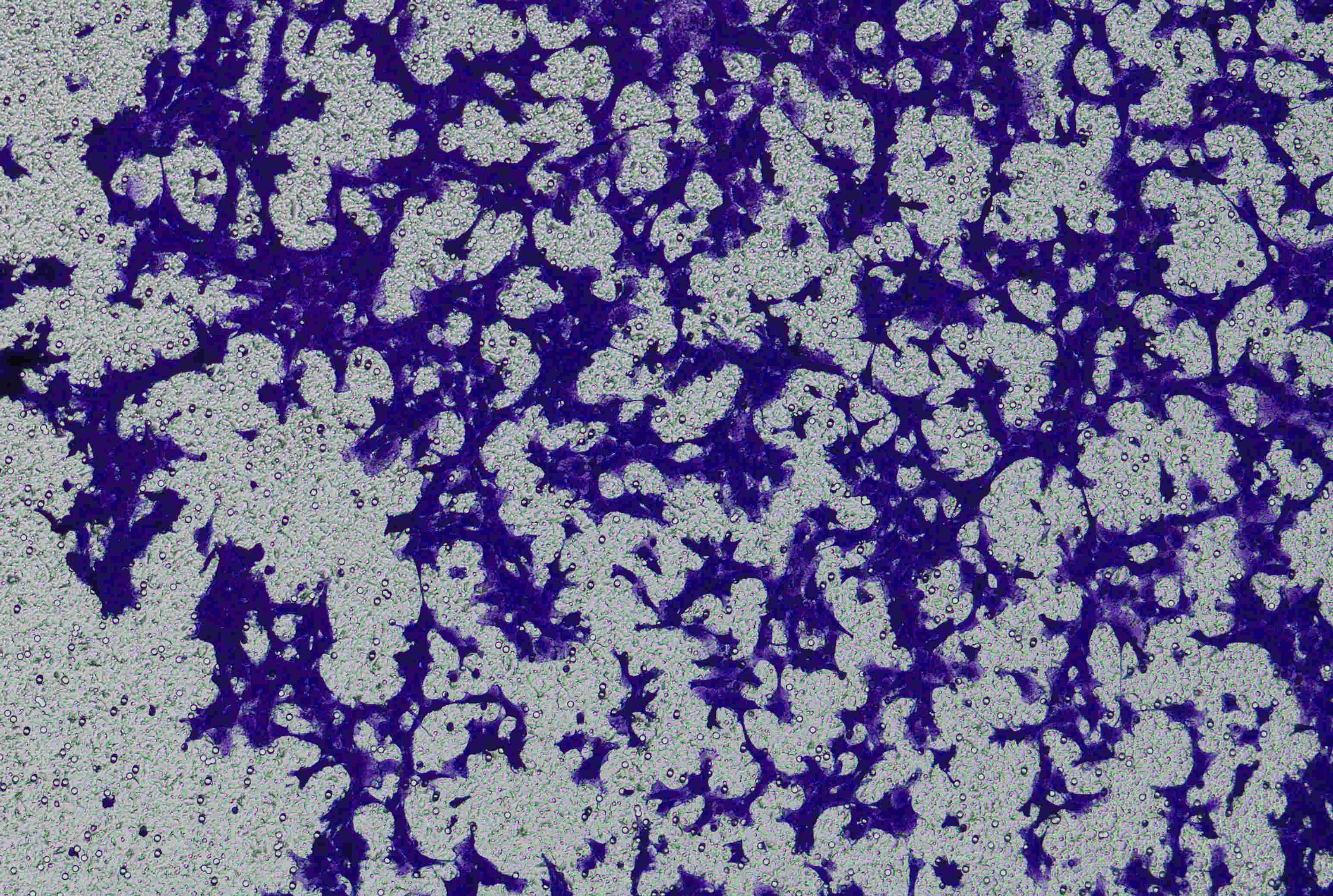

Supplement: Supplementary file 1 [file DataSheet_1.zip › Raw Data/Transwell/T24/INV-NC-140.jpg]

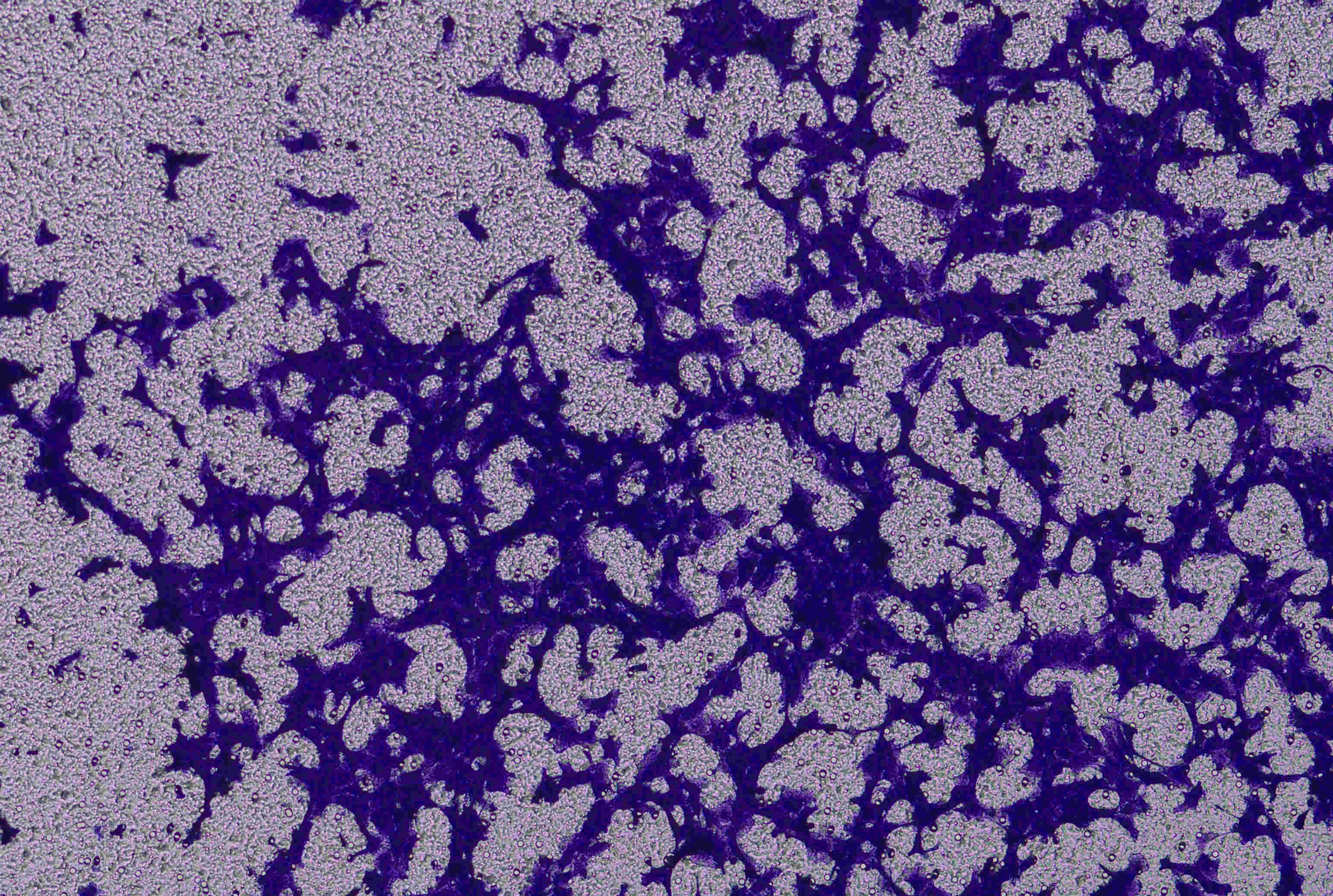

Supplement: Supplementary file 1 [file DataSheet_1.zip › Raw Data/Transwell/T24/INV-NC-141.jpg]

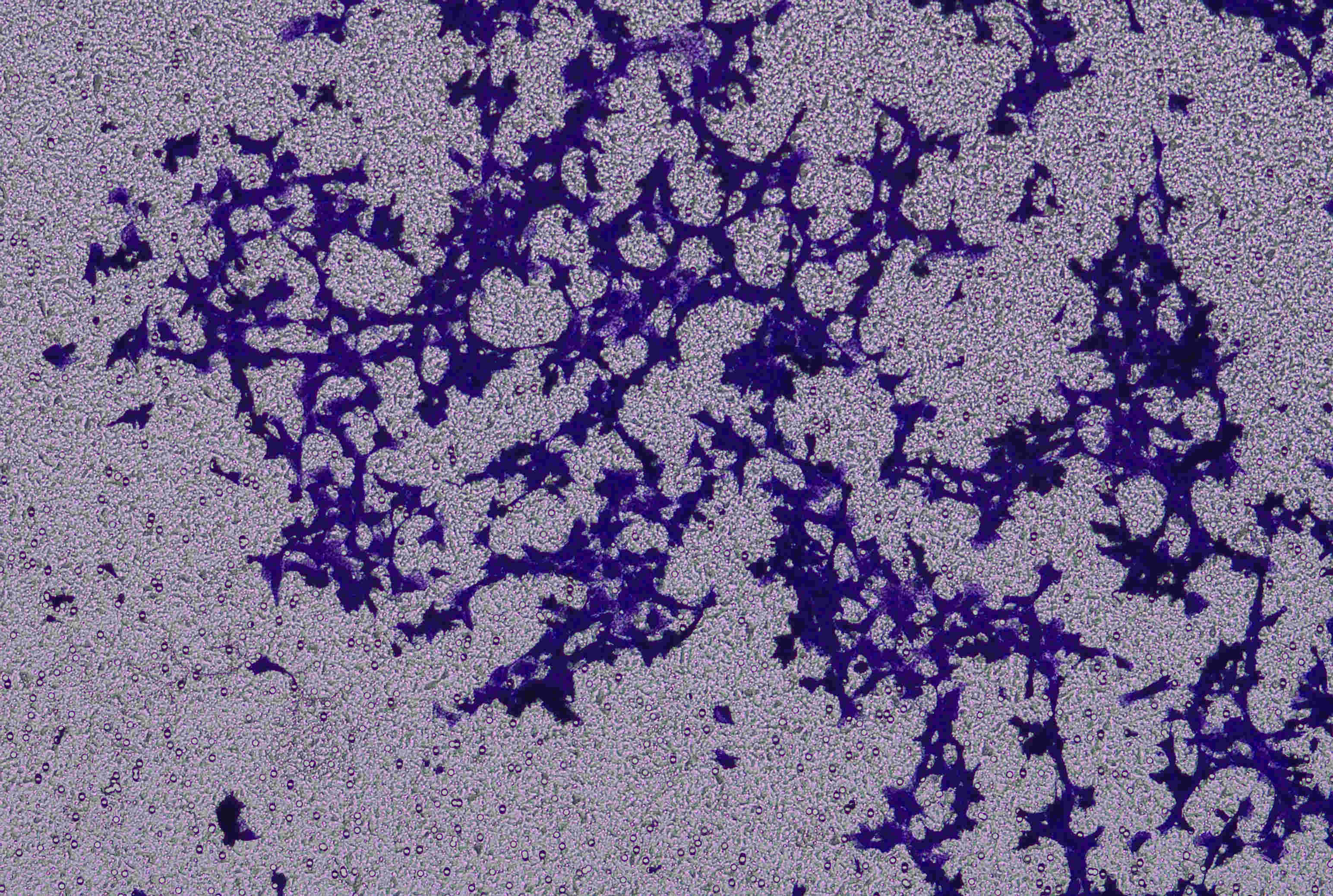

Supplement: Supplementary file 1 [file DataSheet_1.zip › Raw Data/Transwell/T24/MIG-1-101.jpg]

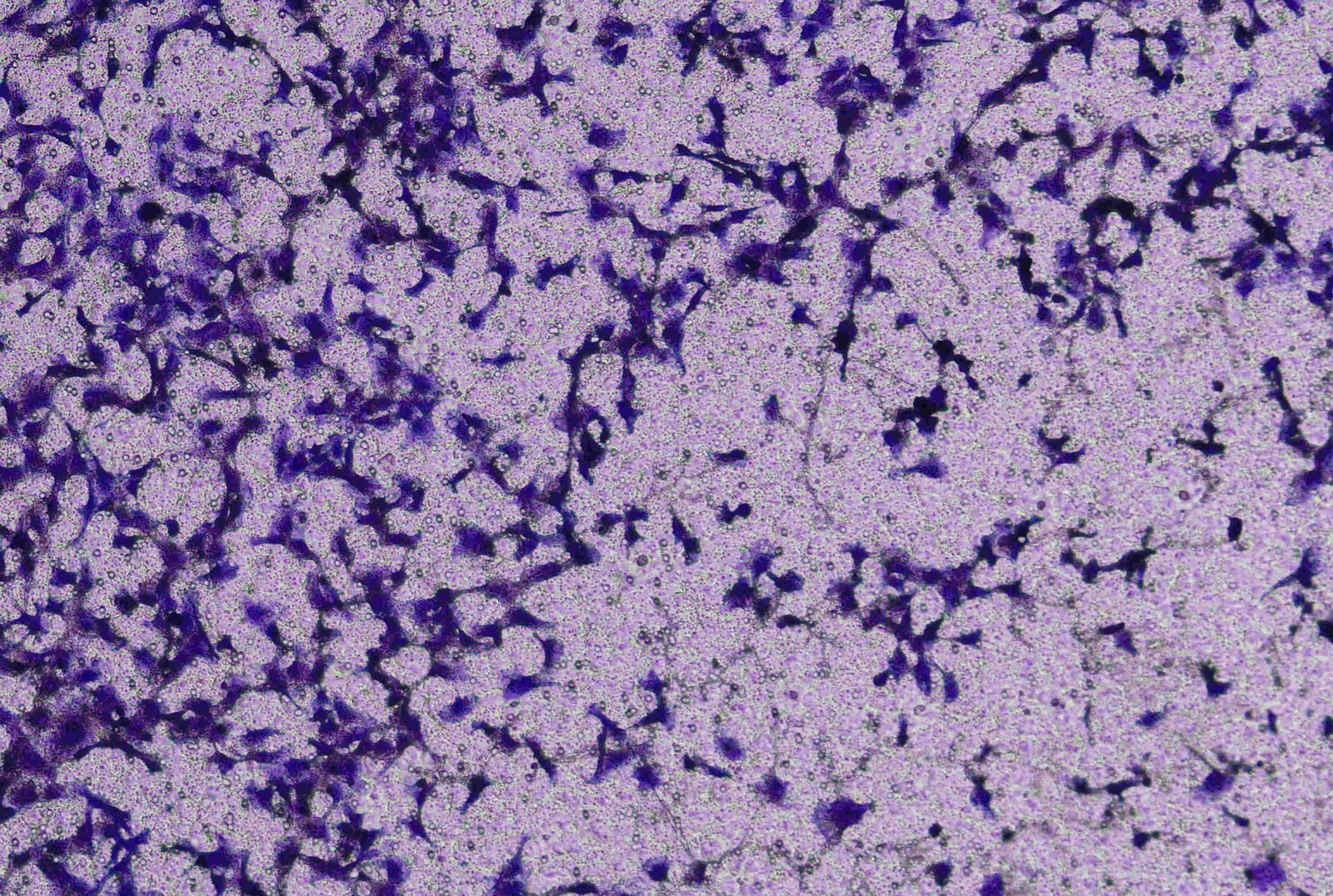

Supplement: Supplementary file 1 [file DataSheet_1.zip › Raw Data/Transwell/T24/MIG-1-112.jpg]

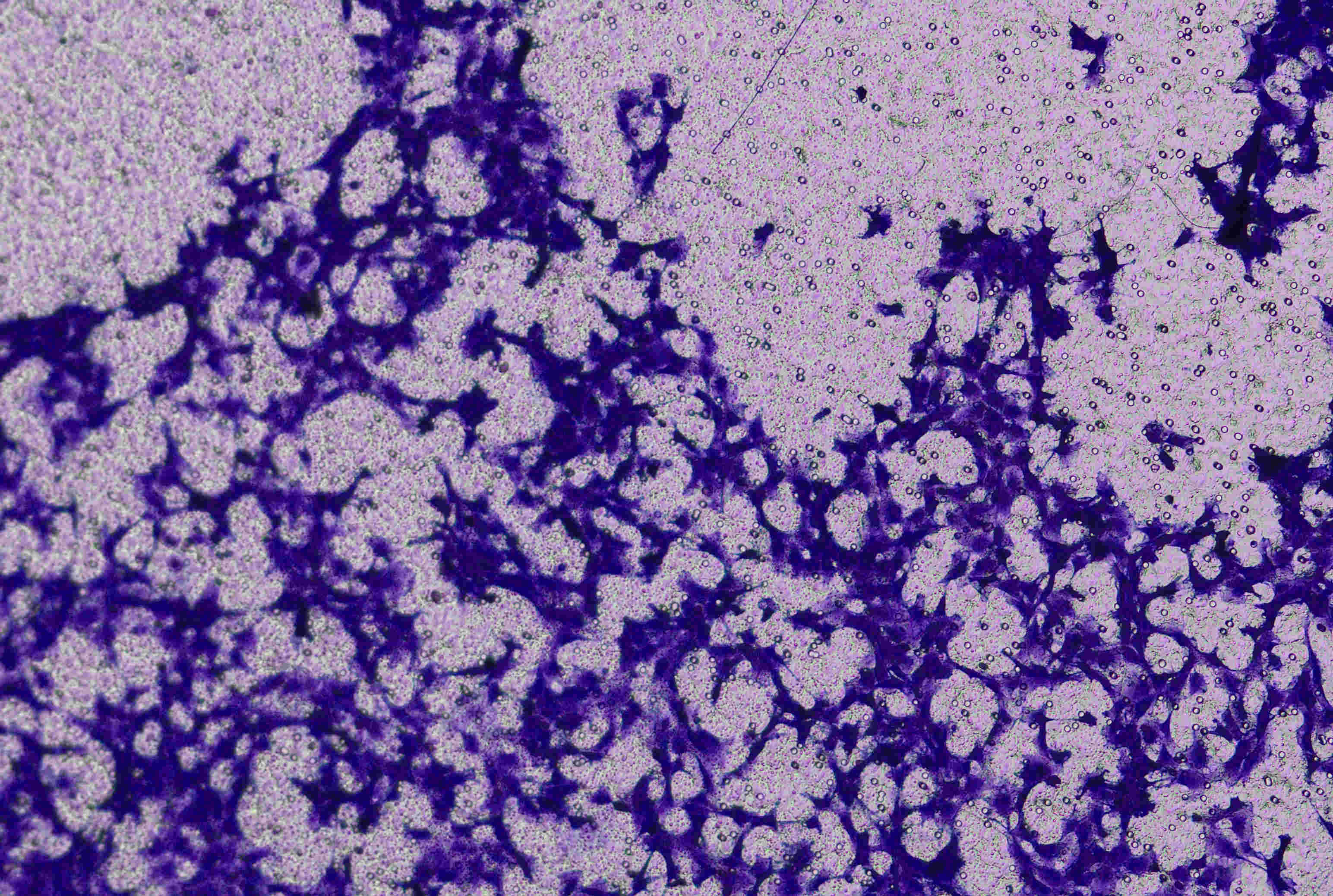

Supplement: Supplementary file 1 [file DataSheet_1.zip › Raw Data/Transwell/T24/MIG-1-120.jpg]

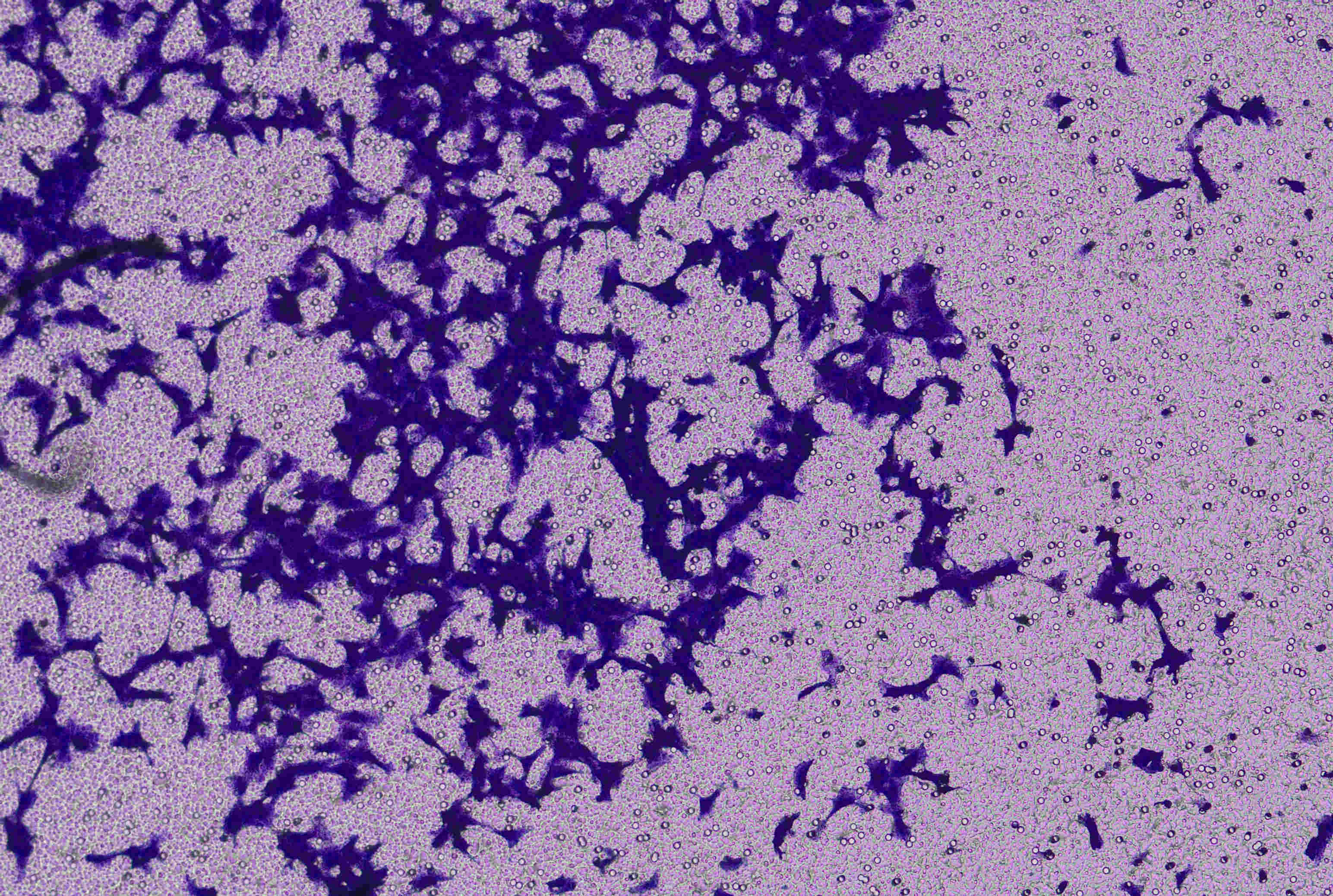

Supplement: Supplementary file 1 [file DataSheet_1.zip › Raw Data/Transwell/T24/MIG-2-115.jpg]

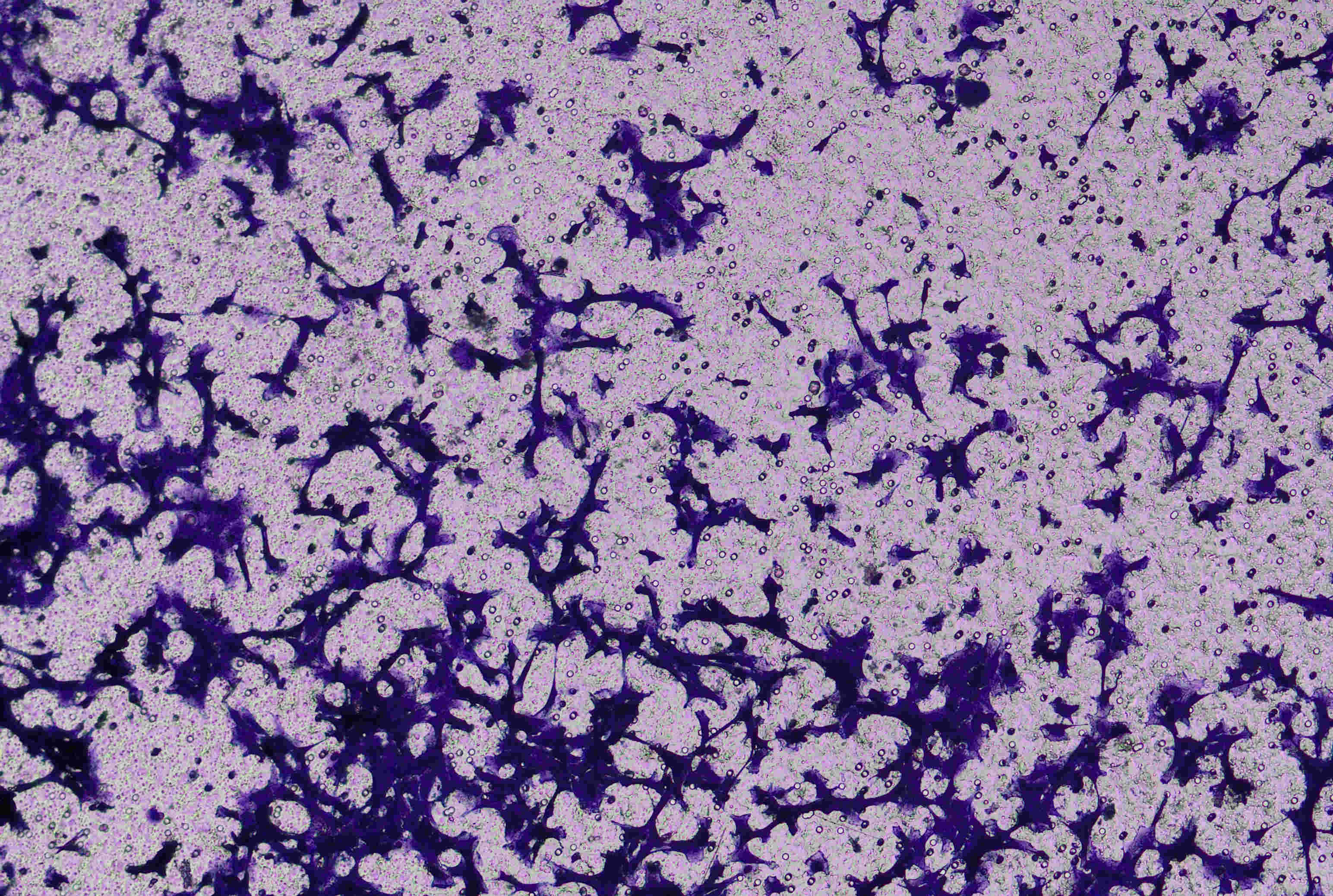

Supplement: Supplementary file 1 [file DataSheet_1.zip › Raw Data/Transwell/T24/MIG-2-123.jpg]

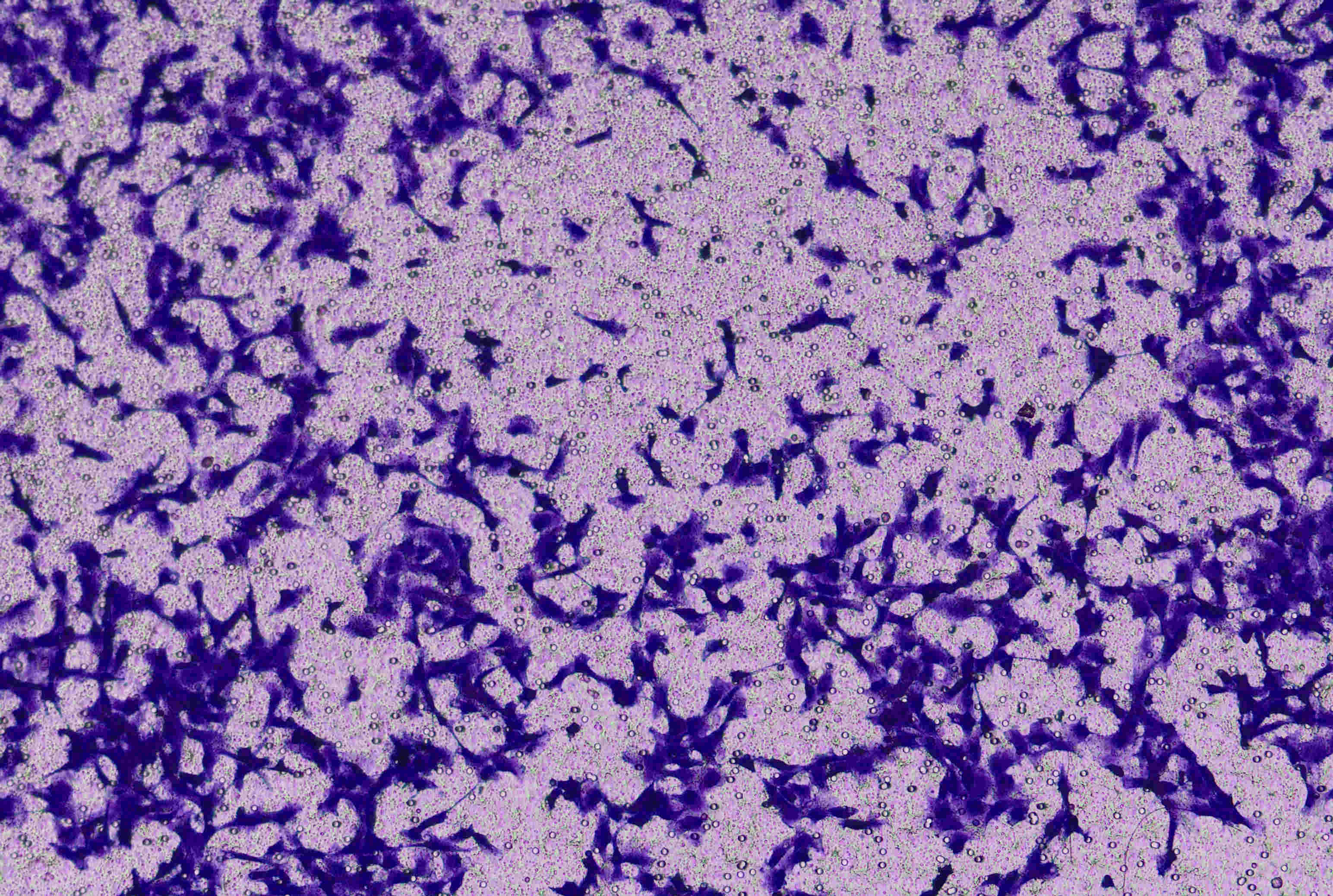

Supplement: Supplementary file 1 [file DataSheet_1.zip › Raw Data/Transwell/T24/MIG-2-128.jpg]

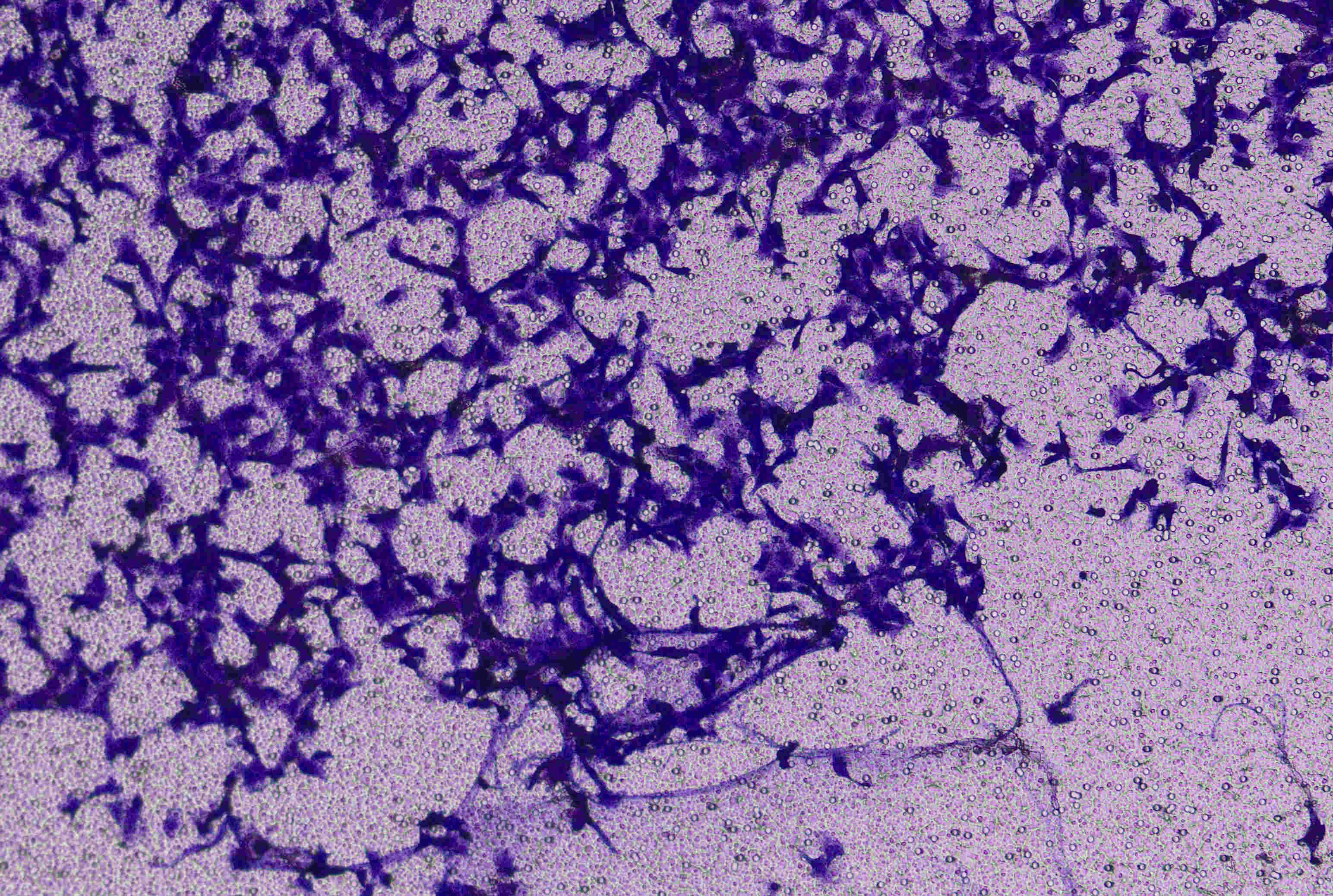

Supplement: Supplementary file 1 [file DataSheet_1.zip › Raw Data/Transwell/T24/MIG-NC-134.jpg]

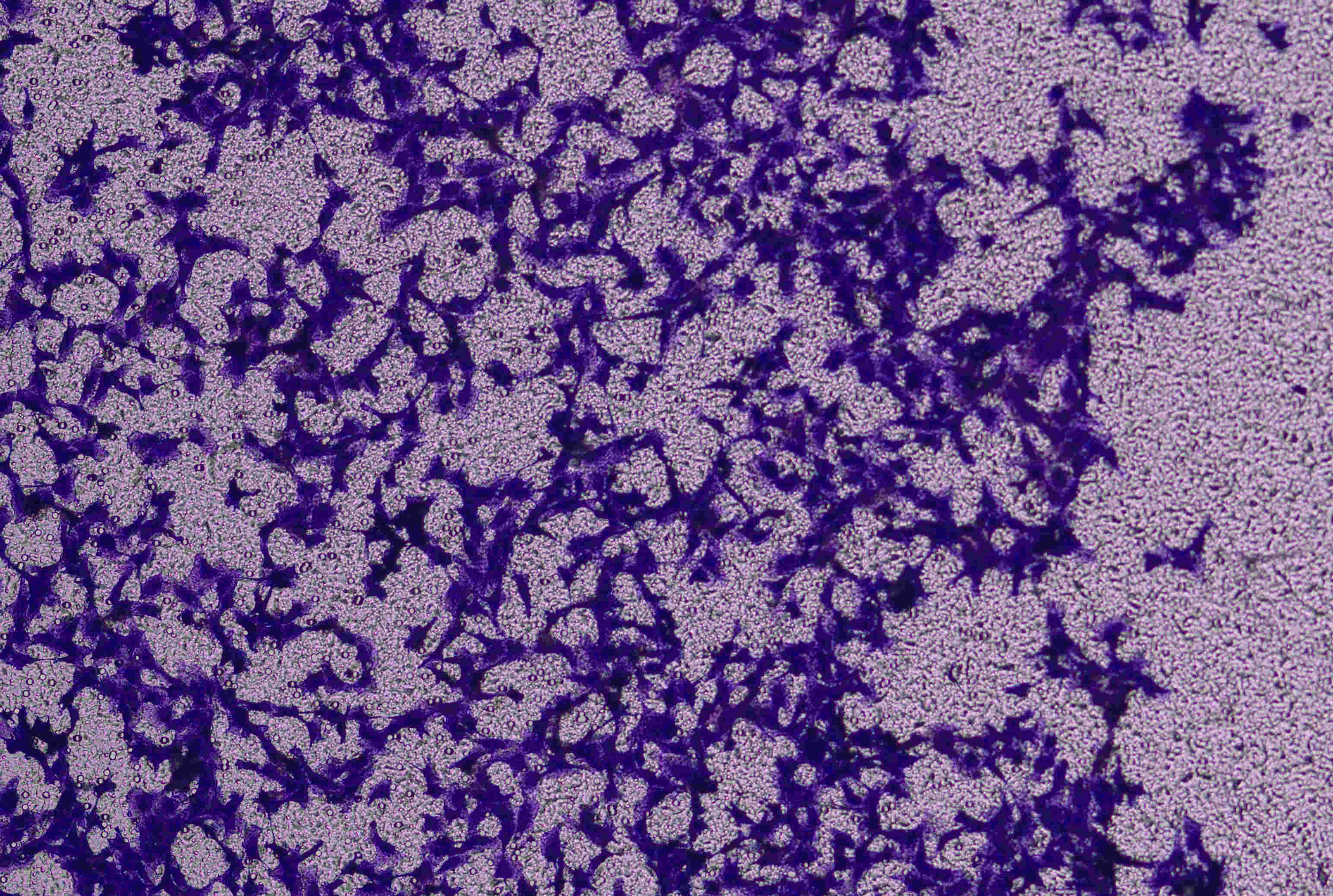

Supplement: Supplementary file 1 [file DataSheet_1.zip › Raw Data/Transwell/T24/MIG-NC-140.jpg]

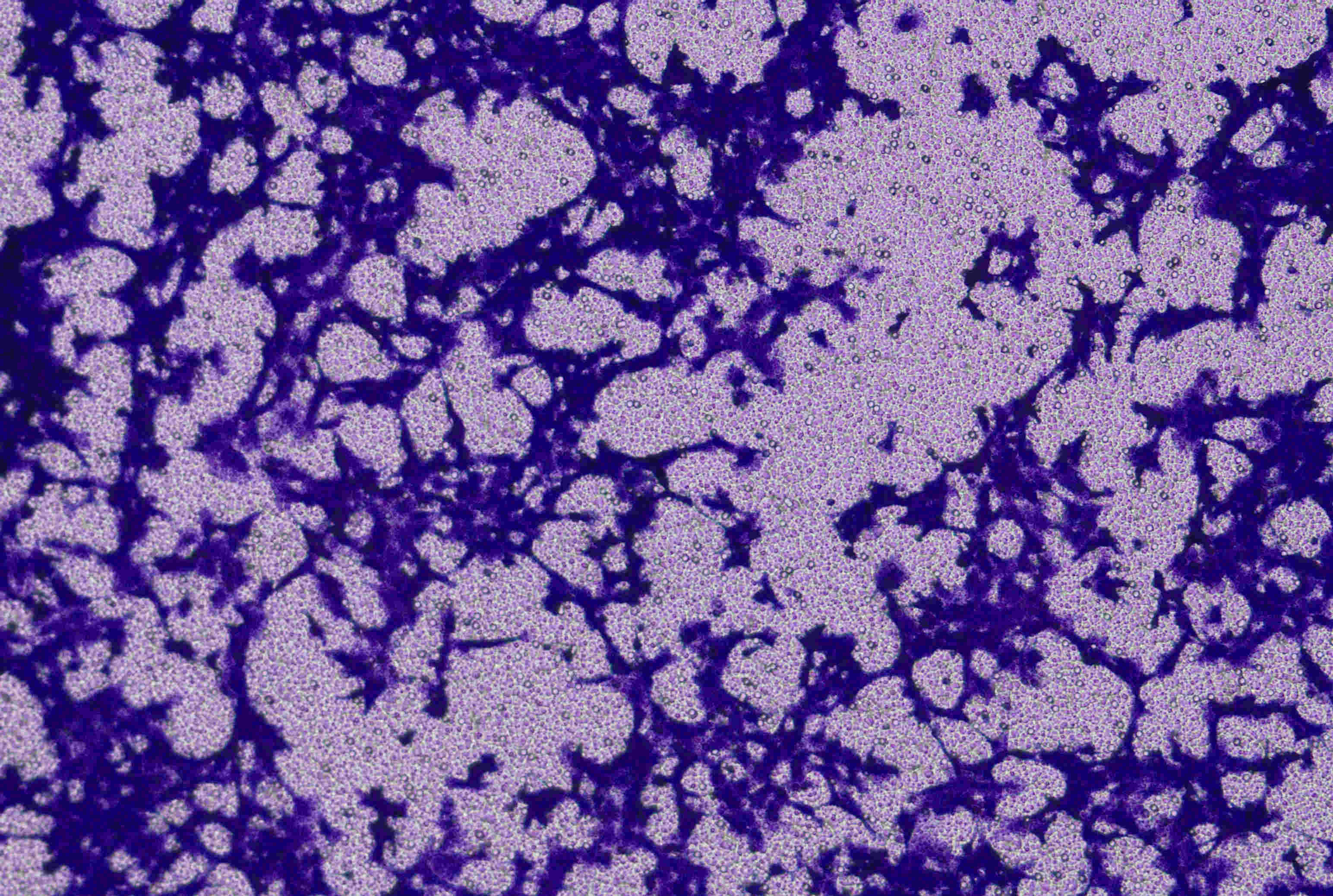

Supplement: Supplementary file 1 [file DataSheet_1.zip › Raw Data/Transwell/T24/MIG-NC-153.jpg]
