# Supplementary material for: Identification of tryptophan metabolism- and immune-related genes signature and prediction of immune infiltration landscape in bladder urothelial carcinoma
Source: Front Immunol. 2023 Oct 26;14:1283792. doi: 10.3389/fimmu.2023.1283792 (PMC10637370; doi:10.3389/fimmu.2023.1283792)
Supplement: Supplementary file 2 [file Table_1.docx]

**Supplementary Table 1. siRNA sequences used in this study.**

| Gene | Sequences (5’-3’) |
| --- | --- |
| si-NAMPT-1 | sense: GCAGAACACAGUACCAUAATT |
|  | antisense: UUAUGGUACUGUGUUCUGCTT |
| si-NAMPT-2 | sense: GGGCCGAUUAUCUUUACAUTT |
|  | antisense: AUGUAAAGAUAAUCGGCCCTT |
| si-NC | sense: UUCUCCGAACGUGUCACGUTT |
|  | antisense: ACGUGACACGUUCGGAGAATT |
