# Supplementary material for: Identification of tryptophan metabolism- and immune-related genes signature and prediction of immune infiltration landscape in bladder urothelial carcinoma
Source: Front Immunol. 2023 Oct 26;14:1283792. doi: 10.3389/fimmu.2023.1283792 (PMC10637370; doi:10.3389/fimmu.2023.1283792)
Supplement: Supplementary file 3 [file Table_2.docx]

**Supplementary Table 2. Primes sequences for RT-qPCR used in this study.**

| **Gene** | **Sequences (5’-3’)** |
| --- | --- |
| NAMPT-forward | CTTCGGTTCTGGTGGAGGTT |
| NAMPT-reverse | CAAAATTCCCTGCTGGCGTC |
| β-actin-forward | GGCGGCACCACCATGTACCCT |
| β-actin -reverse | AGGGGCCGGACTCGTCATACT |
